# Gut microbiota-derived acetate promotes long-term recovery through angiogenesis guided by lymphatic ingrowth in older adults with stroke

**DOI:** 10.3389/fnins.2024.1398913

**Published:** 2024-09-20

**Authors:** Yujia Yuan, Linlin Li, Jingjing Wang, Bat-Otgon Myagmar, Yuxiao Gao, Huan Wang, Zhao Wang, Cong Zhang, Xiangjian Zhang

**Affiliations:** ^1^Department of Neurology, Second Hospital of Hebei Medical University, Shijiazhuang, Hebei, China; ^2^Hebei Collaborative Innovation Center for Cardio-Cerebrovascular Disease, Shijiazhuang, Hebei, China; ^3^Hebei Vascular Homeostasis Key Laboratory for Neurology, Shijiazhuang, Hebei, China

**Keywords:** aging, ischemic stroke, gut microbiota, angiogenesis, lymphangiogenesis, acetate

## Abstract

**Introduction:**

Ischemic stroke is a leading cause of morbidity and mortality in older adults. Therefore, in this study, we sought to understand the interplay between the microbiota, gut, and brain in the context of stroke in older adults.

**Objective:**

To determine whether gut microbiota from younger individuals promotes recovery through angiogenesis in both elderly stroke patients and aged stroke mice, we explored the changes in gut microbiota and the correlation between short-chain fatty acids (SCFAs) and angiogenesis in the aged stroke population. Then, we altered the gut microbiome in aged mice by transplanting microbiota from younger donors before inducing experimental stroke to explore the mechanism by which gut microbiota-derived SCFAs promote angiogenesis.

**Methods:**

Part I: We conducted a single-center, double-blind trial to compare gut microbiota diversity and SCFA levels in fecal samples from older stroke patients with those from younger stroke patients. Additionally, we measured levels of vascular endothelial growth factor (VEGF) and VEGFC levels in plasma to assess their correlation with SCFA levels. Part II: We performed fecal microbiota transplantation (FMT) 3 days before inducing ischemic stroke in aged male mice (16–18) via distal middle cerebral artery occlusion (dMCAO). The FMT was conducted using gut microbiomes from either young donors (2–3 months) or aged donors (16–18 months).

**Results:**

In older stroke patients, gut microbiota diversity was significantly reduced compared to that in younger stroke patients. Furthermore, levels of acetate, a bacterially derived SCFA, were lower and positively correlated with angiogenesis markers (VEGF and VEGF-C). In aged stroke mice, transplantation of young microbiota improved stroke outcomes by promoting angiogenesis, which was facilitated by lymphatic ingrowth into the cortex. This protective effect was linked to gut microbiota-derived acetate, which enhanced lymphangiogenesis by replenishing acetyl coenzyme A.

**Conclusions:**

(a) Gut microbiota-derived acetate promotes angiogenesis post-stroke and (b) lymphatic ingrowth into the cerebral cortex was observed in post-dMCAO mice. These findings suggest that selectively promoting SCFA-producing bacteria, particularly acetate-producers, could be a promising therapeutic strategy to reduce functional impairments in older stroke subjects.

## Highlights

- In older stroke patients, gut microbiota diversity, and acetate concentrations are reduced, with acetate showing a positive correlation with angiogenesis markers (VEGF and VEGF-C).
- Gut microbiota-derived acetate improves stroke outcomes by promoting angiogenesis, which is guided by lymphatic ingrowth in aged stroke mice.
- Transplantation of young microbiota in post-dMCAO mice promotes meningeal lymphangiogenesis and lymphatic ingrowth into cerebral infarction areas.

## Introduction

Over the past decade, it has become increasingly clear that the bidirectional communication between the brain and the gut, known as the microbiota-gut-brain axis (MGBA), has opened new avenues for investigating neurological diseases with the aim of improving prevention and treatment strategies (Crapser et al., 2016; Singh et al., 2016; Stanley et al., 2016; Spychala et al., 2018; Lee et al., 2020a,b). The “top-bottom” communication from the brain to the gut via the MGBA occurs through the autonomic nervous system, which influences gut motility, permeability, and microbiome composition (Singh et al., 2016; Stanley et al., 2016, 2018; Crapser et al., 2016; Durgan et al., 2019; Lee et al., 2020a,b). Conversely, the “bottom-up” signaling from the gut to the brain involves bacterial metabolites, cytokines, and the migration of inflammatory or immune cells (Singh et al., 2016; Wang and Wang, 2016).

Short-chain fatty acids (SCFAs) are key microbial products and represent the end products of gut microbial fermentation of dietary fiber, simple sugars, resistant starch, sugar alcohols, and other indigestible foods in the large intestine (Yao et al., 2022). The main SCFAs—acetate, propionate, and butyrate—constitute approximately 95% of the total SCFA content (Kimura et al., 2020).

All components of the MGBA undergo significant age-related alterations in older adults, including alterations in gut microbiota composition, fecal SCFA levels, intestinal barrier integrity, and central nervous system function (Honarpisheh et al., 2022). Rejuvenation procedures have shown that the microbial community and intestinal immunity of aged mice can be restored to a state comparable to that of young mice (Shin et al., 2021). Moreover, fecal SCFA levels have been observed to decrease with age (Salazar et al., 2019). The function of the intestinal barrier deteriorates with aging, primarily due to a decline in tight junction proteins and mucus production (Parrish, 2017).

Stroke, a common age-related disease, exacerbates these issues by disrupting the gut microbiome, increasing gut permeability, and promoting inflammation (Benakis et al., 2016; Singh et al., 2016; Stanley et al., 2018). Once a stroke has occurred, the crosstalk between the aged gut microbiome and the brain can hinder recovery, which results in poorer outcomes for older adults compared to younger individuals (Crapser et al., 2016). Therefore, studying the relationship between stroke and the gut microbiome is especially significant in the context of aging.

Current stroke treatments, such as intravenous thrombolysis, antiplatelet therapy, and endovascular thrombectomy, primarily target the acute phase of the condition (Herpich and Rincon, 2020). However, long-term recovery after a stroke is equally important and requires serious attention. Neurorestorative processes, including neurogenesis, angiogenesis, and synaptic plasticity, are critical for recovery (Beck and Plate, 2009). Angiogenesis, the natural process of forming new blood vessels, is particularly crucial for restoring oxygen and nutrient supply to ischemic brain tissue. This process involves the growth of new capillaries through sprouting and splitting from existing vessels (Wang et al., 2017; Kanazawa et al., 2019; Myagmar et al., 2022).

Studies have shown that SCFAs promote proangiogenesis in the mesentery and during granulation tissue formation (Moghadamrad et al., 2015; Castro et al., 2021). Interestingly, one of the many changes associated with aging is a decrease in SCFA-producing bacteria and SCFAs themselves (Nagpal et al., 2018; Spychala et al., 2018). The beneficial effects of SCFAs have been observed in organs outside the gut, as they can circulate through the bloodstream (Moghadamrad et al., 2015; Lee et al., 2020a,b). For example, probiotics and SCFAs, which regulate microglia function in the brain, play an important role in the development of the blood–brain barrier and improve post-stroke recovery and cognitive performance (Braniste et al., 2014; Lee et al., 2020a,b). However, it is still unclear whether gut microbiota-derived SCFAs have similar proangiogenic effects in the context of cerebral ischemia.

Young animals usually recover quickly during the acute phase of stroke, which does not fully replicate the complicated effects of ischemia on the brain in older hosts. Thus, it is important to study aged animals to better target conditions that are most relevant to the older stroke population. To this end, we conducted studies by analyzing stroke cases in older individuals through clinical sample analysis and animal experiments in aged mice (16–18 months).

Given the context, we hypothesized that the gut microbiota and SCFA levels in the older stroke population would differ from those in the younger stroke population, and we performed correlation analyses between gut microbiota-derived SCFAs and angiogenic factors. Additionally, we tested the hypothesis that microbiota and their metabolites from young gut microbiota could promote proangiogenesis in the brains of aged mice. If altering the unhealthy gut microbiome proves effective, this research could pave the way for novel approaches to stroke prevention and treatment in the older population.

## Materials and methods

### Part I: Exploring changes in gut microbiota and the correlation between SCFAs and angiogenesis in aged stroke humans

#### Clinical trial and participants

We conducted a single-center, double-blind trial to compare gut microbiota and SCFA levels in fecal samples from older stroke patients with those from younger stroke patients. Additionally, we measured VEGF and VEGFC levels in plasma to assess their correlation with SCFA levels.

The study included 37 stroke patients aged ≥ 65 (Lin et al., 2024; Si et al., 2024) who were admitted to the Neurology Department of the Second Hospital of Hebei Medical University between September 2022 and September 2023. This group was recruited as the case group (old). The control group (Ctrl) with 43 stroke patients younger than 65 years (Lin et al., 2024; Si et al., 2024) was also recruited from the same department during the same period. In older adults, the age range was from 67 to 81 years, with a mean ± SD of 71.65 ± 4.461 years. The control group ranged in age from 31 to 63 years, with a mean ± SD of 52.86 ± 8.758 years.

The Research Ethics Committee of the Second Hospital of Hebei Medical University approved the study (approval letter no. 2022-R733). The trial was registered at http://www.chictr.org.cn (clinical registration number: ChiCTR2200065052).

The inclusion criteria included the following: (1) patients who met the diagnostic criteria for stroke, confirmed by brain computed tomography (CT) or magnetic resonance imaging (MRI); (2) all patients who experienced their first-ever stroke, with inclusion occurring 7–15 days after the onset of stroke; (3) patients who were aged 18 to 85 years at the time of inclusion; (4) patients who had been living in Hebei Province for more than 3 years and for more than 10 months per year; (5) patients who had no special dietary habits; and (6) patients or the authorized principals who signed informed forms.

The exclusion criteria included the following: (1) patients who had other neurological diseases; (2) patients who suffered from digestive system diseases, autoimmune diseases, circulatory diseases, and respiratory diseases within the past 3 months; (3) patients who had taken probiotics, prebiotics, antibiotics, proton pump inhibitors (PPI), mesalazine, laxatives, sex hormones, oral contraceptives, antidepressants, opioids, and metformin within the past month; (4) patients who were unable to cooperate or suffering from conditions that would interfere with behavioral assessments and treatment; (5) patients who were suffering from life-threatening severe diseases; and (6) pregnant or lactating women.

#### Study design

All patients received standard clinical treatments, including antiplatelet drugs, statins, blood pressure and glucose control, and other pharmacological therapies. Additionally, all patients received rehabilitation treatments, such as physical therapy and acupuncture therapy.

Demographic and clinical data were collected, including age, sex, height, weight, body mass index (BMI), tobacco use, excessive alcohol consumption, time since onset, blood pressure, high-density lipoprotein cholesterol (HDL-C), low-density lipoprotein cholesterol (LDL-C), triglyceride (TG), total cholesterol (TC), total bile acid (TBA), homocysteine (Hcy), degree of culprit vessel stenosis (Li et al., 2023), National Institutes of Health Stroke Scale (NIHSS) score, and modified Rankin Scale (mRS) score.

For sample collection, 1 g of fresh feces was collected from each participant, placed in a sterile tube containing DNA preservation solution, and stored at −80°C. Additionally, 2 ml of peripheral blood samples were collected from each participant. After coagulation, the serum was separated by centrifugation at 4,000 g for 15 min and stored at −80°C for further analysis.

#### Gut microbiota 16S rRNA gene sequencing

Fecal samples were transported by dry ice to Majorbio BioPharm Technology Co., Ltd. (Shanghai, China) for analysis. According to the manufacturer's instructions, microbial DNA was extracted from the fecal samples using the E.Z.N.A. Soil DNA Kit (Omega Bio-Tek, Norcross, GA, USA). The DNA concentrations were measured using a NanoDrop 2000 UV-vis spectrophotometer (Thermo Scientific, Wilmington, DE, USA), and DNA quality was evaluated using 2% agarose gel electrophoresis.

The V3–V4 region of the 16S rRNA gene was sequenced using the Illumina MiSeq platform. Specific primers, 338F (5′-ACTCCTACGGGAGGCAGCAG-3′) and 806R (5′-GGACTAC HVGGGTW TCTAAT-3′) were used to amplify this hypervariable region. PCR was performed in a thermocycler (GeneAmp^®^ 9700; ABI, Foster City, CA, USA) under the following conditions: initial denaturation at 95°C for 3 min, followed by 27 cycles at 95°C for 30 s, annealing at 55°C for 30 s, elongation at 72°C for 45 s, and a final extension at 72°C for 10 min. Each PCR assay was conducted in triplicate in a 20-μl reaction mixture containing 4 μl 5 × FastPfu Buffer, 2.5 mmol/L dNTPs (2 μl), 0.8 μl of each primer (5 μmol/L), 0.4 μl FastPfu polymerase, and 10 ng template DNA.

The PCR products were separated on a 2% agarose gel, purified using the AxyPrep DNA Gel Extraction Kit (Axygen Biosciences, Union City, CA, USA), and quantified using the QuantiFluor™-ST system (Promega, Madison, WI, USA) following the manufacturer's instructions. After splicing, QIIME2 (2022.2) was used to process the raw reads and to remove low-quality sequences. Operational taxonomic units (OTUs) were defined for sequences with over 97% similarity. The purified amplicons were then pooled in equimolar concentrations and subjected to paired-end sequencing (2 × 300 bp) on an Illumina MiSeq system (Illumina, San Diego, CA, USA)^®^.

#### Determination of SCFA contents in mice

The contents of SCFAs, including acetate, propionate, butyrate, isobutyrate, valerate, isovalerate, caproate, and heptanoic acid, were measured in feces, serum, and brain tissue. Fecal samples (50 mg), serum samples (100 μl), and brain tissue (100 mg) were spiked with 40 μl of a methanol/water (4:1, v/v) solution (Merck, Darmstadt, Germany) and extracted according to the manufacturer's instructions (Majorbio Bio-Pharm Technology Co., Ltd., Shanghai, China). The extracted samples were evaluated using liquid chromatography-mass spectrometry (LC-MS) (Shimadzu, Tokyo, Japan). Aliquots from all extracted samples were pooled to create a quality control (QC) sample, which was processed and analyzed in the same manner as the analytical samples.

#### Measurement of VEGF and VEGFC in blood samples by ELISA

The concentrations of VEGF and VEGFC in serum were measured using commercially available ELISA kits: Human VEGF Quick ELISA Kit (96T, Boster Biotech Co., Ltd., China, FEK0539), Human VEGF-C ELISA Kit (96T, Boster Biotech Co., Ltd., China, EK0588), and Mouse VEGF ELISA Kit (96T, Boster Biotech Co., Ltd., China, FEK0541). All incubation and washing steps were performed following the manufacturer's recommended protocols.

Briefly, the serum samples were added to enzyme wells that had been pre-coated with specific antibodies. Subsequently, a recognition antigen labeled with horseradish peroxidase (HRP) was added to each well. After incubation for 30 min at 37°C, the HRP-labeled antigen competed with the solid-phase antigen, forming an immune complex. The HRP enzyme then catalyzed the conversion of tetramethyl benzidine to a blue color, which subsequently turned yellow upon interaction with an acid. The absorbance was measured at a wavelength of 450 nm using a multifunctional enzymatic instrument (Tecan, Switzerland). The concentrations of VEGF and VEGFC were determined by comparing the absorbance values to a standard curve.

### Part II: Exploring the mechanism of gut microbiota-derived SCFAs in promoting angiogenesis in aged stroke mice

#### Animals and dMCAO models of stroke

Male C57BL/6 mice (16–18 months old, weighing 30–35 g, 2–3 months old, weighing 21–23 g; RRID:IMSR_CRL:27) of specific pathogen-free (SPF) grade were purchased from Vital River (Beijing Vital River Laboratory Animal Technology, China). The animals were group-housed under SPF conditions, maintained on a 12/12 h light/dark with 60% ± 5% humidity and a temperature of 22°C ± 3°C, with free access to food and water.

All animal care and experimental procedures were conducted in strict accordance with the regulations for the Care and Use of Laboratory Animals published by the National Institutes of Health (NIH) and the ARRIVE guidelines for reporting experiments (Kilkenny et al., 2010; McGrath et al., 2010). All animal procedures were approved by the Animal Care and Management Committee of the Second Hospital of Hebei Medical University (Permit No. HMUSHC-130318). Every effort was made to minimize animal suffering and the number of animals used. All surgeries and experiments were performed by investigators who were blinded to the experimental group assignments.

Focal cerebral cortical ischemia was induced through the permanent occlusion of the middle cerebral artery (MCA) and the common carotid artery (CCA). The animals were anesthetized with an intraperitoneal injection of Avertin (400 mg/kg, Sigma-Aldrich, Cat#T48402-25G). An approximate 1 cm median incision was made in the neck to carefully isolate and permanently ligate the right common carotid artery using a 3–0 silk surgical suture. Subsequently, a second incision was made between the right eye and ear, and a small hole approximately 2 mm in diameter was drilled into the skull using a high-speed dental drill (Microdrill, 0.5 mm burr; RWD Life Science, China) to expose the right distal MCA. Finally, the distal MCA was coagulated with a cauterizer (Bovie, USA) under a microscope so as not to damage the brain surface. Throughout the procedure, the body temperature of the mice was maintained at 37.5°C ± 0.5°C using a heating pad and a heat lamp. The detailed procedures for dMCAO have been previously described (Caballero-Garrido et al., 2015).

#### Fecal microbiota transplantation

Mice were orally gavaged with streptomycin sulfate (500 mg, Solarbio, Cat. No. S8290) in 50 μl sterile for the first 2 days before the fecal transplant gavage (FTG). To assess the reduction of bacterial load after streptomycin treatment, fecal samples (Abx group) were collected before FTG. FMT was conducted following an established protocol (Borody et al., 2013).

Briefly, feces from naïve young or aged mice (*n* = 5–10 per group) were collected under sterile conditions. A total of 0.1 g of fresh feces from donor mice were pooled and suspended in 1 ml of sterile ice-cold PBS. The solution was vigorously mixed for 15 s using a benchtop vortex (Vortex-Genie 2, Scientific Industries, USA; speed 9). The suspension was then centrifuged at 800 g for 3 min at room temperature, and the supernatant was collected. This supernatant was administered to dMCAO mice via oral gavage (500 μL/mouse) for the first 3 days before dMCAO induction.

#### Experimental design

In these experiments, mice were randomly assigned to one of two groups: the young-FTG group (mice that received dMCAO and FMT from young, healthy mice) and the old-FTG group (mice that received dMCAO and FMT from old mice). To detect dividing cells, 5-Bromo-2-deoxyuridine (BrdU, Sigma-Aldrich Cat# T48402) was used. In some experiments, mice were intraperitoneally injected with 50 mg/kg/day of BrdU starting 24 h after dMCAO and continuing until the animals were euthanized.

**Figure 1:**
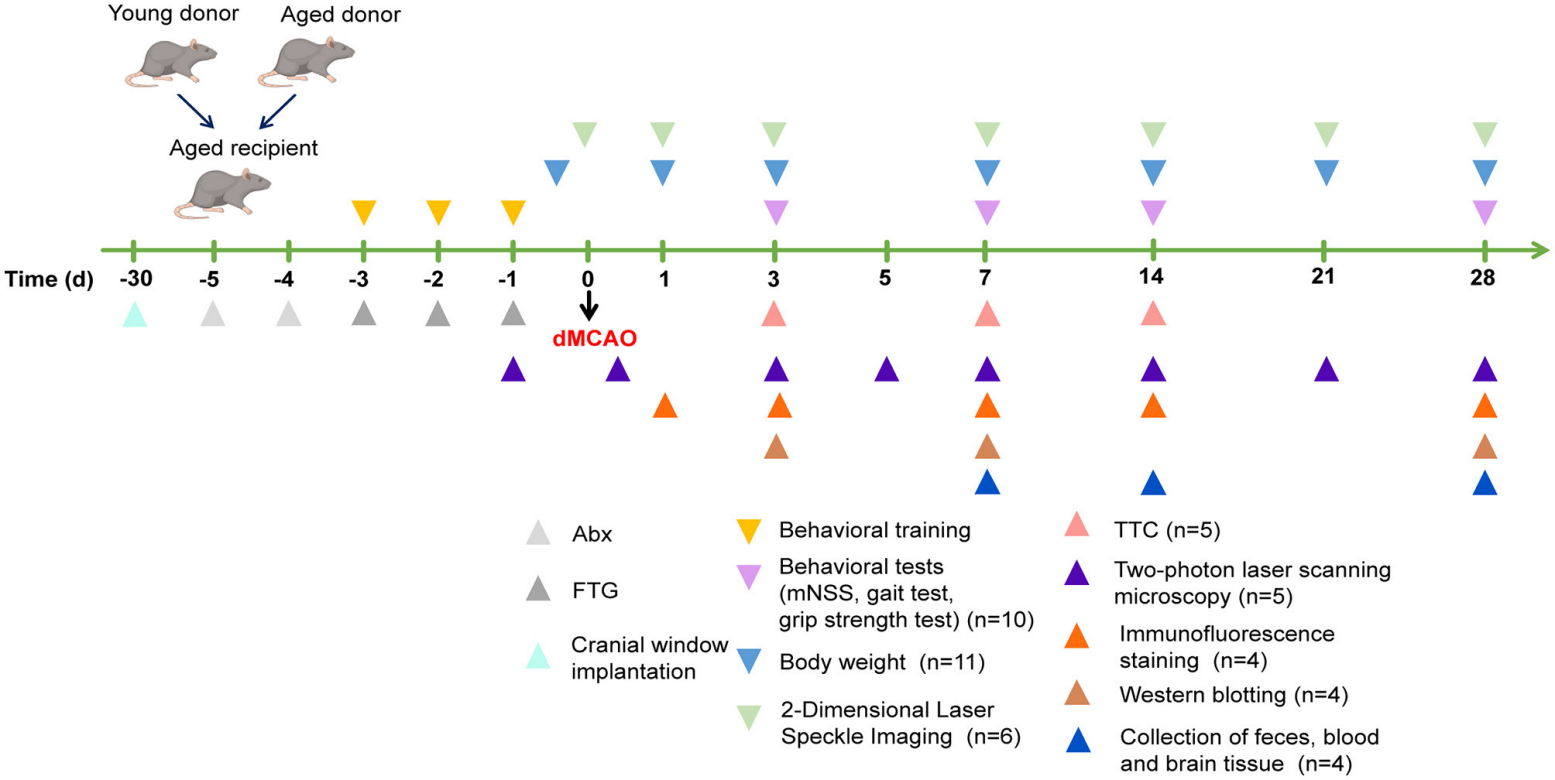
The experimental specific flow chart of experimental procedures and date.

The mice were euthanized by rapid decapitation under deep anesthesia on days 3, 7, 14, and 28 after cerebral ischemia, according to the experimental design, and samples were collected for further analysis. The specific experimental flow chart is shown in Figure 1.

#### Collection of feces, blood, and brain tissue

To collect feces, the mouse was gently restrained by pinching the neck with the left hand while holding the tail with the right hand. Fecal pellets (3–5) were collected directly into sterile EP tubes without touching the anus. Then, the collected feces were frozen and stored at −80°C.

After anesthetizing the mice, they were placed on their backs, and the chest area was shaved and disinfected. Blood was collected by inserting a needle at the junction of the xiphoid process and the left costal arch, slowly withdrawing blood. After collection, pressure was applied with sterile gauze to stop the bleeding. The blood samples were centrifuged at 4,000g at 15°C for 10 min, and the supernatant was collected. Both the serum and brain tissue were stored at −80°C until further use.

The detailed steps for gut microbiota 16S rRNA gene sequencing, determination of SCFA contents, and ELISA are listed above.

#### Behavioral tests

##### mNSS

The modified neurological severity score (mNSS) is a composite evaluation tool that assesses motor, sensory, balance, and reflex functions, with scores ranging from 0 to 18 (where 0 represents normal function and 18 represents maximal deficit) (Cao et al., 2020). A higher score indicates worse neurological function. All neurological evaluations were recorded by an investigator who was blinded to the group assignments.

##### Gait test

TreadScan (v2.0 CleverSys, USA), a gait analysis system, was used to measure footfalls and motor performance in ischemic mice (Caballero-Garrido et al., 2017). Every mouse was allowed to walk freely through a corridor on a glass walkway, which was illuminated from below. A camera was used to record the movements of all four paws at a rate of 100 frames/sec for 20 s. The recorded data were then analyzed using TreadScan software (v4.00). A successful walking test was defined as the mouse walking at a steady speed without stopping, rearing, or grooming).

Before undergoing MCAO, the mice in each group were trained for 3 consecutive days to familiarize them with the test. During the actual test, mice were placed at the maximum speed at which they could maintain coordinated locomotion. Each mouse was tested thrice, with at least a 15-min interval between trials. For gait analysis, the stride length of the left front and rear limbs was measured on days 1, 7, 14, and 28 post-stroke.

##### Grip strength test

The grip strength test measures the animal's maximum force when grabbing a bar. To ensure reliable assessment using the Grip Strength Meter (Bioseb, France), it is crucial that the mice are accustomed to being handled. Prior to the study, mice were acclimated and trained with the grip strength test (Vaysse et al., 2015). During the test, when the unrestrained forepaw was brought into contact with the bar, the mouse instinctively grasped the bar to prevent falling, while the experimenter gently pulled away from the device.

The handling and training process lasted 3 days to ensure that the experimenter could consistently reproduce the pulling strength. This consistency was confirmed when similar grip strength values were obtained for both paws, with a measurement variation of <10% before dMCAO. Following this preparation, strength testing (three trials per paw per session) was carried out on days 1, 7, 14, and 28 after dMCAO.

##### Brain infarct volume

The infarct volume was evaluated using 2,3,5-triphenyl tetrazolium chloride (TTC) staining on days 3, 7, and 14 after dMCAO. Mice were deeply anesthetized, after which their brains were rapidly removed and frozen at −20°C for 20 min. The brains were then sliced into five coronal sections, each approximately 1 mm thick, and stained with 2% TTC solution (Sigma-Aldrich, St. Louis, MO, USA) at 37°C for 30 min. After staining, the sections were fixed in 4% paraformaldehyde for 24 h. An observer, blinded to the experimental groups, calculated the infarct volume using NIH ImageJ software (National Institutes of Health, RRIDSCR_003070). Infarct volumes were corrected for edema and determined using the following equation (Bederson et al., 1986):


$$\begin{array}{l} Infarct\;volume\;(\%)=[Total\;Infarct\;Volume\;-\\ \;(Right\;Hemisphere\;Volume\;-\\ \;Left\;Hemisphere\;Volume)]Left\;\\ \;Hemisphere\;Volume\;\times \;100\%. \end{array}$$

#### Laser speckle contrast imaging *in vivo*

Laser speckle contrast imaging is used for full-field and depth-integrated imaging of blood flow dynamics, providing reliable spatial perfusion indices that are valuable for characterizing vascular changes after ischemic stroke (Ponticorvo and Dunn, 2010; Schrandt et al., 2015). Cortical surface cerebral blood flow (CBF) dynamics were quantified using a laser speckle blood flow monitoring video system (Peri Cam PSIHR, PSIH-10129, Peri Med AB, Sweden) along with the PIMSoft system (Software Version I**·**S, Peri Med AB, Sweden).

Under anesthesia, the mice were shaved and disinfected on the calvaria. A median incision of approximately 1.5 cm was made. Then, the mice were placed on a stereotaxic frame (Narishige Scientific Instrument Lab, Tokyo, Japan) to immobilize the head, and CBF was recorded through a cranial window. Consistent settings for zoom, gain, and pseudo-color thresholds were maintained throughout the experiment. CBF measurements were recorded at baseline and on days 0, 1, 3, 7, 14, 21, and 28 after dMCAO.

#### Two-photon laser scanning microscopy *in vivo*

Two-photon laser scanning microscopy provides a depth-resolved technique for monitoring the degree and time scale of post-stroke vascular structures in three dimensions (Schrandt et al., 2015). Before imaging, the mice were prepared with a closed cranial window. The mice were first placed in an isoflurane induction chamber connected to an isoflurane vaporizer, with the isoflurane levels set at 4% at a rate of 1 L/min. Under continuous anesthesia delivered via a mask, each mouse was positioned on a stereotaxic frame (Narishige Scientific Instrument Lab, Tokyo, Japan) to immobilize the head, and erythromycin eye ointment was applied to protect the eyes.

The calvaria was shaved and disinfected, with the skin over the calvaria carefully removed. The craniotomy region was selected based on stereotactic coordinates, located 3 mm caudal and 3 mm lateral to bregma in the intended lesioned hemisphere. This region partially overlaps peri-infarct zones, as confirmed by MRI performed 24 h poststroke (Tian et al., 2023). Then, a dental drill (Microdrill, 0.5 mm burr; RWD Life Science, China) was used to thin the edges around the 4-mm^2^ craniotomy region. The bone within the craniotomy region was then carefully removed while keeping the dura intact.

A custom-made cover glass was created by gluing together 3-mm and 5-mm round cover glasses (Warner Instruments, Sarasota, FL, USA) using UV-cured optical glue (5,800 ergo, Metal One-component instant cyanoacrylate adhesives, Switzerland). Superglue (Loctite) was then applied to stick the cover glass to the edge of the craniotomy region. Once the glue had dried, a custom-made head plate was attached to the contralateral side of the skull using dental cement (Monomer 10 ml, Polymer 5 g, Super Bond C&B, Sun Medical, Japan).

After surgery, the mice were returned to their cages for recovery. Dexamethasone (2 mg/kg, intramuscular) was administered to reduce the immune response. Additionally, antibiotics and carprofen were provided for 7 consecutive days to prevent infection and reduce pain days. After cranial window implantation, the mice were given 2 to 4 weeks to recover.

Under anesthesia, 70-kDa fluorescein isothiocyanate–dextran (Fluorescein isothiocyanate-dextran, SLCC2644, Sigma Aldrich, USA) was intravenously injected via the tail vein into recipient mice 15 min before imaging to demarcate the vasculature. LSM 880 (Zeiss, Germany) was used for 2-photon live imaging. A vascular image was captured by scanning an area of 425.1 μm × 425.1 μm with a pixel size of 0.83 μm and a frame size of 512 × 512 pixels, and the images were processed using the ZEN 2.1 SP3 system (Germany). The scanning speed was set to 7. A laser wavelength of 800 nm, excited by a MaiTai laser at a power of 5% to 10%, was used for 2-photon imaging. Images of 512 × 512 pixels were acquired. Z stacks were scanned to a depth of 200 to 300 μm through the closed cranial window. The diameter and density of the pial collaterals were then measured for analysis. Images were recorded at baseline and on days 0, 3, 5, 7, 14, 21, and 28 after dMCAO.

#### Exfoliation of dural lymphatic vessels in mice

The mice were anesthetized with Avertin and then perfused transcardially with saline, immediately followed by cold 4% paraformaldehyde in 0.1 M phosphate-buffered saline (PBS). To begin the dissection, we made an incision at the junction of the skull and atlas, separating the skin and muscles to expose the intact skull. Finally, a circular cut was made from the foramen magnum along the base of the parietal bone, allowing for the careful removal of the intact top of the skull. After the skull was immersed in EDTA (0.5 M, pH = 7.4) at 4°C for 24 h, the dural membrane was carefully peeled off under a stereomicroscope and then attached to pathological slides. The slides were allowed to dry at room temperature before further use.

#### Immunofluorescence staining

Mice were anesthetized with Avertin and perfused transcardially with saline, quickly followed by cold 4% paraformaldehyde in 0.1 M phosphate-buffered saline (PBS). The brains were then dehydrated in 30% sucrose for 48 h. Frozen coronal brain sections were cut into 15 μm slices using a cryotome (Thermo Scientific, USA). The sections were permeabilized with 0.3% Triton X-100 for 20 min.

In addition, for BrdU-labeled sections, the brain slices were incubated with 2 N HCL at 37°C for 30 min and then washed with boric acid (pH 8.5) at room temperature. Then, the sections were blocked with 10% normal donkey serum for 1 h at 37°C and then incubated with primary antibodies overnight at 4°C. The primary antibodies used were rabbit anti-CD31 (1:100, Abcam Cat# ab222783, RRID:AB_2905525), rat anti-Lyve-1 (1:100, Santa Cruz Biotechnology, Cat# sc-65647, RRID:AB_1123635), sheep anti-bromodeoxyuridine (BrdU, 1:200, Abcam Cat# ab1893, RRID:AB_302659), rabbit anti-occludin antibody (1:400, Protein Technology, Cat# 27260-1-AP, RRID:AB_2880820), and rabbit anti-Claudin-1 antibody (1:100, Bioworld Technology, Cat# BS1063, RRID:AB_1664011).

The following day, the slices were washed with PBS and incubated with the appropriate corresponding secondary antibodies (Alexa Fluor 488 or 594, Jackson Immuno Research, USA) at 37°C for 1 h. Color images were captured using a laser scanning confocal microscope (Zeiss LSM880, German). Microvessel density and BrdU+/CD31+ cell counts were analyzed using ImageJ software.

#### Western blotting

Protein from the colon and cortex was extracted using Radio Immunoprecipitation Assay (RIPA) lysis buffer (Solarbio Cat# R0020) supplemented with 1% protease inhibitor cocktail (Sigma Cat# P8340) and 1% phosphatase inhibitor (Applygen Cat# P1260). The protein concentrations in the supernatant were quantitatively measured using a bicinchoninic acid protein assay reagent kit (Thermo Fisher Scientific, USA). Equivalent amounts of protein (50 μg) were separated by SDS-PAGE and transferred onto a polyvinylidene fluoride (PVDF) membrane (Roche, USA).

The membranes were then blocked with protein-free rapid blocking buffer (Epizyme Cat# PS108) in Tris-buffered saline containing 0.1% Tween-20 for 30 min at room temperature. Followed blocking, the membranes were incubated overnight at 4°C with the following primary antibodies: rabbit anti-occludin antibody (1:5000, Protein Technology, Cat# 27260-1-AP, RRID:AB_2880820), rabbit anti-Claudin-1 antibody (1:500, Bioworld Technology, Cat# BS1063, RRID:AB_1664011), rabbit anti-VEGFA antibody (1:500, ABclonal Technology, Cat# A5708, RRID:AB_2766467), rabbit anti-Angiopotein-1 antibody (1:500, Boster Biological Technology, Cat# PB0092), rabbit anti-VEGFC antibody (1:500, Boster Biological Technology, Cat# BA0548), rabbit anti-Lyve-1 antibody (1:500, Boster Biological Technology, Cat# BM4905), rabbit anti-PROX1 antibody (1:500, Abcam, Cat# ab199359, RRID:AB_2868427), rabbit anti-Beta actin antibody (1:5000, Bioworld Technology, Cat# AP0060, RRID:AB_2797445), and rabbit anti-GAPDH antibody (1:10000, Bioworld Technology, Cat# AP0063, RRID:AB_2651132).

After washing the membranes three times with TBST, they were incubated with Goat Anti-Rabbit IgG (H&L) Antibody DyLight 800 Conjugated (1:10000, Rockland Cat# 611-145-122, RRID:AB_1057618) for 1 h at room temperature. The relative density of each band was detected using an Odyssey infrared scanner (LICOR Bioscience, USA), and the intensity of the bands was analyzed using ImageJ software.

#### Statistical analysis

Statistical analyses and graph generation were conducted using GraphPad Prism software (version 9.0). All data were tested for normality and variance homogeneity. Continuous data following a normal distribution were described using means and percentages. The median and interquartile range (IQR) were used to describe continuous variables with skewed distributions, while categorical variables were summarized as numbers and percentages.

When the normality test was satisfied, statistical comparisons between the two groups were conducted using either Student's *t-*test or a two-way ANOVA. For non-parametric data, the Wilcoxon rank sum test (Man–Whitney *U-*test) was used. All statistical analyses and graph generation were performed using GraphPad Prism software (version 9.0).

For correlation analyses, the Pearson correlation coefficient was calculated for data following a bivariate normal distribution, while the Spearman correlation coefficient was used for data that did not follow a bivariate normal distribution. All statistical tests were two-sided, with a *p-*value of <0.05 considered statistically significant. In the statistical analysis, ^*^ indicates a *p*-value of <0.05, ^**^ indicates *a p*-value of <0.01, and ^***^ indicates a *p*-value of <0.001.

A multivariate statistical analysis was conducted using Bioconductor on the Majorbio Cloud Platform (https://cloud.majorbio.com). Based on OTU cluster analysis, both statistical and visual analysis of gut microbiota at the phylum and genus levels were performed using R Studio (4.1.3) (Schloss et al., 2009). Alpha diversity was assessed using the Wilcoxon rank sum test, with a *p*-value of <0.05 considered significantly different. Beta diversity was evaluated using the Bray–Curtis algorithm and analyzed using the Analysis of Similarity (ANOSIM) test.

To compare the relative abundance of significantly different taxa in the gut microbiota, the researchers employed linear discriminant analysis effect size (LEfSe), where an LDA value of >2 was considered an essential contributor to the model. The correlation between OTU relative abundances and demographic and clinical data was analyzed using Spearman's correlation. Differential OUTs between the two groups were mapped to their biochemical pathways through metabolic enrichment, and pathway analysis was conducted using the Kyoto Encyclopedia of Genes and Genomes (KEGG) database (http://www.genome.jp/kegg/). These metabolites were classified according to their associated pathways and functions.

## Results

### Part I: In older stroke patients, the diversity of gut microbiota and acetate concentration decrease, and acetate positively correlates with angiogenesis markers (VEGF and VEGF-C)

#### Characteristics of the microbiota in older stroke patients compared to younger patients

We first profiled the gut bacterial composition of the two groups (old, ctrl) using 16S rRNA sequencing. The results revealed significant differences in the diversity and composition of gut bacteria between aged and young stroke patients.

**Figure 2:**
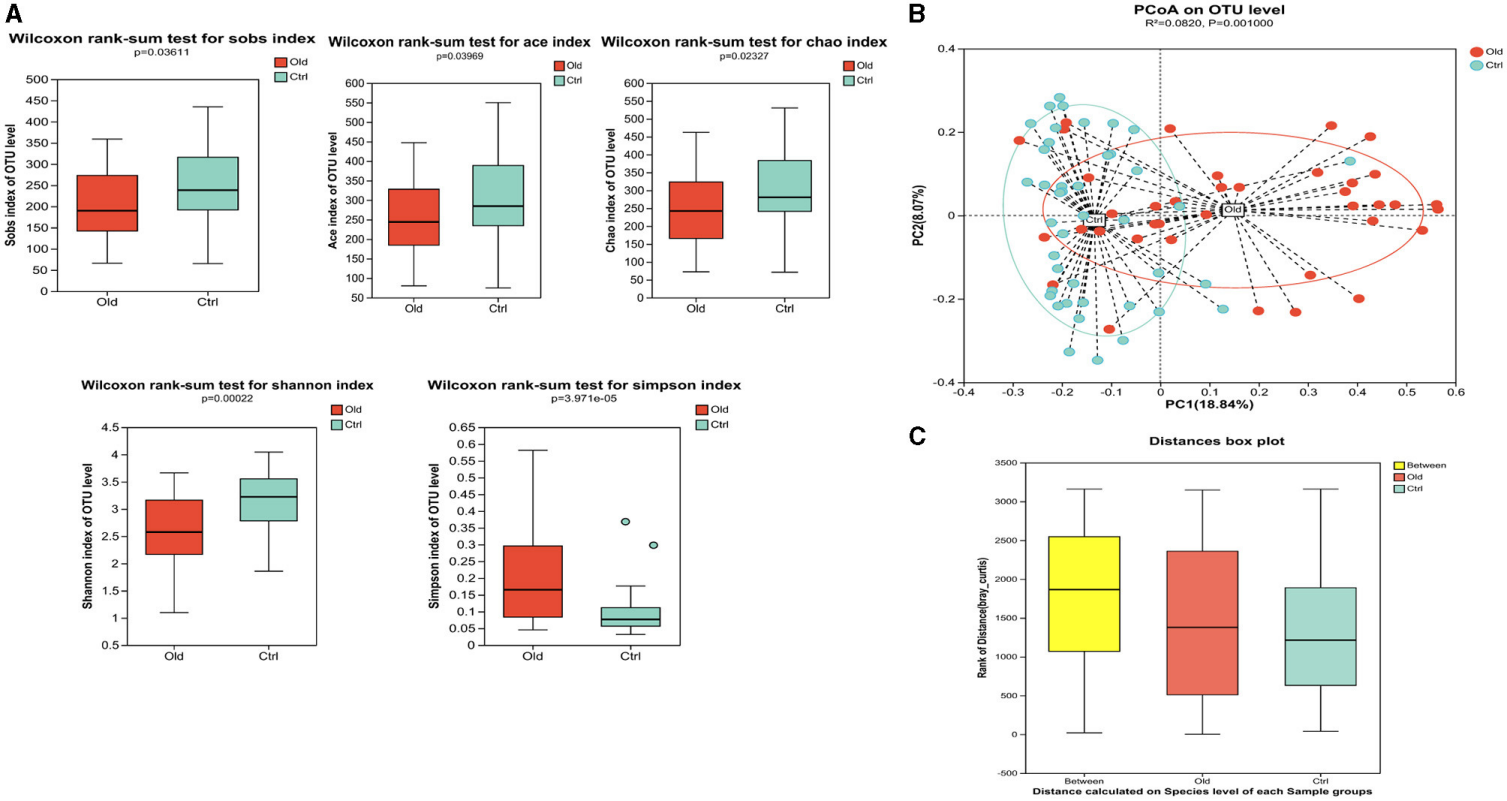
Gut microbiota characteristics in older stroke patients compared to young stroke patients. **(A)** Alpha diversity analysis. The analysis revealed statistically significant differences in species abundance and diversity between the two groups. The Sobs, Chao1, and Ace indices showed statistically significant differences (*P* = 0.036, *P* = 0.040, *P* = 0.023, respectively). The Shannon index showed that the diversity in the old group was lower than in the ctrl group (*p* < 0.05). The Simpson index showed the evenness in the ctrl group was superior to that in the old group (*p* < 0.05). **(B)** Beta Diversity Analysis (PCoA Plot at OTU Level). The Principal Coordinate Analysis (PCoA) plot at the OTU level showed a clear separation between the old and control groups, indicating distinct differences in microbiota composition. **(C)** ANOSIM test showed that the differences between the groups were significantly greater than the differences within groups.

In terms of Alpha diversity, the aged group's gut bacteria community exhibited significantly lower abundance (as measured by Sobs, Chao1, and Ace indices), diversity (Shannon index), and evenness (Simpson index) compared to the young group (Figure 2A).

Principal coordinate analysis (PCoA) was used to assess the β-diversity between the two groups, which was based on Bray–Curtis algorithms [R = 0.0820, P = 0.001 (ANOSIM test)], showing significant differences in the microbiota community structure (Figures 2B, C).

To ensure the samples accurately represented the bacterial community, we evaluated the α diversity of the Sobs index at the OTU level and examined the rarefaction curve for both Sobs and Shannon indices (Supplementary Figure S1). These analyses confirmed that the samples were appropriate and reasonable for representing the microbiota community.

**Figure 3:**
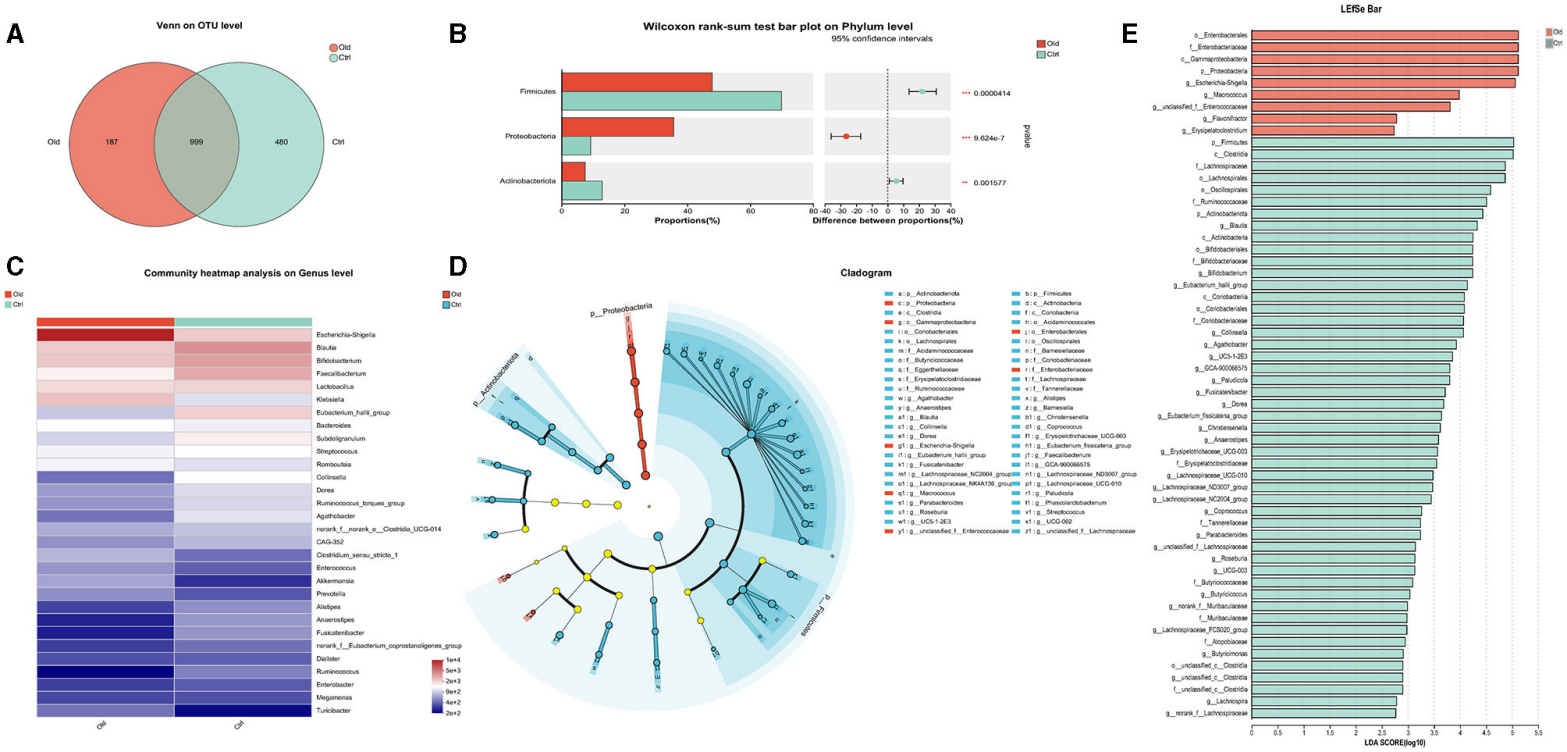
The different taxonomical levels of distribution of gut microbiota between the old and Ctrl groups. **(A)** Venn diagram. The Venn diagram illustrates the number of common and unique characteristic OTUs (Operational Taxonomic Units) among the two groups. It highlights the shared and distinct OTUs present in the gut microbiota of older and young stroke patients. **(B)** Phylum-level differences. This panel shows significant alterations in taxa at the phylum level between the two groups, indicating shifts in the overall composition of gut microbiota. **(C)** Genus-level heatmap. The heatmap visualizes the relative abundances of the 30 most predominant bacteria at the genus level across the two groups. It provides a comparative view of how these bacterial populations differ between older and younger stroke patients. **(D)** LEfSe cladogram. The LEfSe (linear discriminant analysis effect size) cladogram displays the dominant bacteria in the two groups. The diameter of each circle represents the abundance of the corresponding bacteria, while the color indicates its phylogenetic level. Different colored nodes signify microbial groups that are significantly enriched in either the Old or Ctrl group, while yellow nodes indicate no significant difference between the groups. **(E)** LEfSe bar chart. The LEfSe bar chart identifies key differences in bacterial abundance from the phylum to genus level between the Old and Ctrl groups. Only taxa with a significant LDA (Linear Discriminant Analysis) threshold value of >2 are shown, highlighting the most critical differences in bacterial populations between the two groups.

The taxonomical distribution of gut microbiota was shown as follows. The Venn diagram showed a total of 1666 OTUs, with a total of 999 common OTUs in all two groups, 187 unique OTUs in the Old group, 480 unique OTUs in the Ctrl group (Figure 3A). We drew Bar and Circos diagram to intuitively show the change trend of species in different groups (Supplementary Figures S2A, B). Specifically, the phylum *Proteobacteria* was significantly more abundant in the aged stroke patients than in the young (35.63% and 9.26%, respectively, q <0.05), and the abundance of *Firmicutes* and *Actinobacteriota* were lower in the aged stroke patients compared with the young stroke patients (47.93% vs. 69.92%, 7.53% vs. 12.92%, respectively, q <0.05) (Figure 3B). In the Old group, the most abundant population at genus level was *Escherichia-Shigella*, followed by *Klebsiella, Bifidobacterium*, and *Blautia* (Figure 3C). In the Ctrl group, the top four most abundant were *Blautia, Bifidobacterium, Faecalibacterium* and *Lactobacillus* (Figure 3C). LEfSe showed that the bacterial communities of the Old group had a lower abundance of *Faecalibacterium, Blautia, Bifidobacterium, Eubacterium, Collinsella, Butyricicoccus*, et al. and had a higher abundance of *Escherichia-Shigella, Macrococcus, Enterococcaceae, Macrococcus*, and *Erysipelatoclostridium* compared with the Ctrl group (Figures 3D, E). Together the results showed above that aged stroke patients' fecal microbiota was less diverse and compositionally different from young stroke patients'. Together, these results indicate that the fecal microbiota of older stroke patients is less diverse and compositionally distinct from that of young stroke patients.

**Figure 4:**
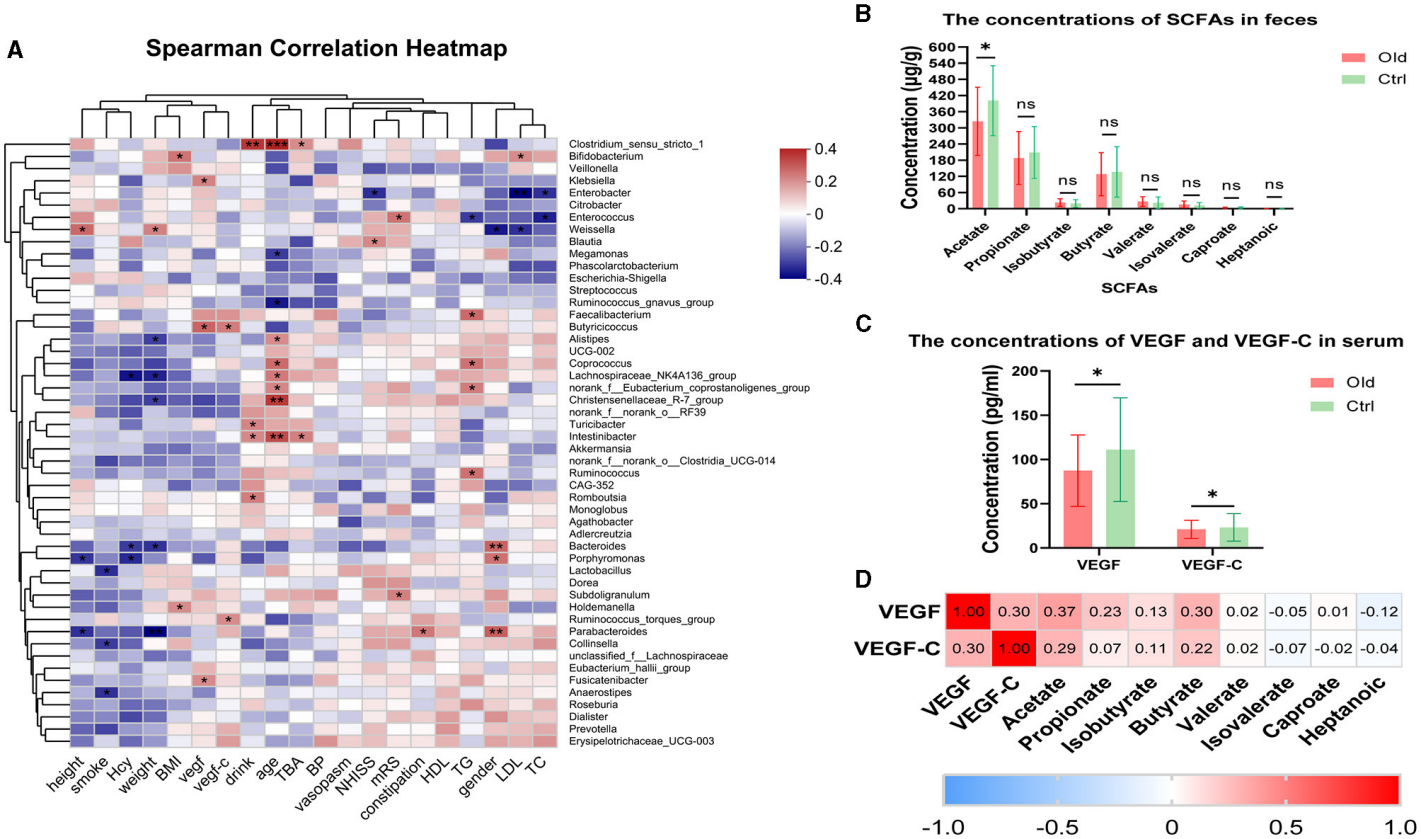
**(A)** Heatmap of Spearman rank correlation analysis between gut microbiota and clinical indexes in the Old and Ctrl groups at the genus level. Red indicates a positive correlation, and blue indicates a negative correlation. *P < 0.05, **P < 0.01, ***P < 0.001 for comparisons between the Old and Ctrl groups. **(B)** Graph comparing individual SCFA levels between the Old and Ctrl groups. *P*-values are as follows: *P* = 0.0246, *P* = 0.3254, *P* = 0.2444, *P* = 0.7661, *P* = 0.1540, *P* = 0.1777, *P* = 0.0557, and *P* = 0.1344 for acetate through heptanoic acid, respectively. **(C)** Comparison of VEGF and VEGF-C levels in serum between the two groups. **(D)** Spearman correlation analysis among VEGF, VEGF-C, and SCFAs. Red indicates a positive correlation, and blue indicates a negative correlation. The value in each cell represents the correlation coefficient (r). Acetate, propionate, isobutyrate, and butyrate showed varying degrees of positive correlation with VEGF and VEGF-C, while the other SCFAs did not.

Correlation analyses between the 50 most abundant bacterial communities and clinical indices were conducted in both the Old and Ctrl groups using Spearman correlation (*P* < 0.05) (Figure 4A). We found numerous interesting correlations at the genus level (Figure 4A).

Notably, blood pressure and vasospasm were not closely related to the stroke patients' gut microbiota in the trial. However, other clinical indices showed positive or negative correlations with the gut microbiota. The strongest correlation was between age and specific microbiota. *Lactobacillus* and *Collinsella* were negatively correlated with smoking. Additionally, *Clostridium_sensu_stricto* showed a strong positive correlation with both age and excessive drinking. Interestingly, *Ruminococcus, Butyricicoccus*, and *Fusicatenibacter*, which are common SCFA producers, were positively associated with VEGF and VEGF-C levels.

#### Acetate concentration decreases in older stroke patients and positively correlates with VEGF and VEGF-C

We measured the concentrations of fecal primary metabolites, including SCFAs such as acetate, propionate, isobutyrate, butyrate, valerate, isovalerate, caproate, and heptanoic acid, in both the Old and Ctrl groups. Mass spectrometry analysis revealed an inverse relationship between age and SCFA concentrations, with a significant decrease in acetate levels observed with aging (*P* = 0.0246) (Figure 4B). Moreover, we measured VEGF and VEGF-C levels in serum, which showed significant differences between the two groups (VEGF: *P* = 0.0166, VEGF-C: *P* = 0.0334) (Figure 4C).

We found that levels of acetate, VEGF, and VEGF-C all decreased with age in stroke patients, prompting us to investigate potential correlations between SCFAs and these angiogenesis markers. Notably, acetate and butyrate were positively correlated with VEGF (*r* = 0.372 and *P* = 0.001, *r* = 0.30 and *P* = 0.007, respectively; Figure 4D). Similarly, VEGF-C showed a positive correlation with both VEGF (*r* = 0.30, *P* = 0.006) and acetate (*r* = 0.29, *P* = 0.010) (Figure 4D).

These data prompted us to test whether SCFAs could be a viable prevention method for reducing functional impairments in older adults by promoting angiogenesis in ischemic brain tissue. However, controlling for variables such as dietary habits, genotype, stroke severity, and other confounding factors in the human population was not feasible. To explore this more clearly, we conducted experiments on aged male mice.

### Part II: Gut microbiota-derived acetate improves stroke outcomes by promoting angiogenesis and lymphatic ingrowth in aged stroke mice

#### Recipient-aged stroke mice replicate the gut bacterial profiles of the donor mice

The details of the experimental protocol for FMT and follow-up procedures are shown in the “Materials and Methods” section and illustrated in “Figure 1.” Aged male mice (16–18 months) were gavaged with streptomycin (StM) for 2 consecutive days to reduce their existing bacterial load, facilitating more efficient colonization by the newly introduced bacteria (Supplementary Figure S3). After the streptomycin treatment, FMT was performed using microbiota from either normal young mice (2–3 months; Young-FTG) or normal aged mice (16–18 months; Aged-FTG) for 3 consecutive days before dMCAO induction.

**Figure 5:**
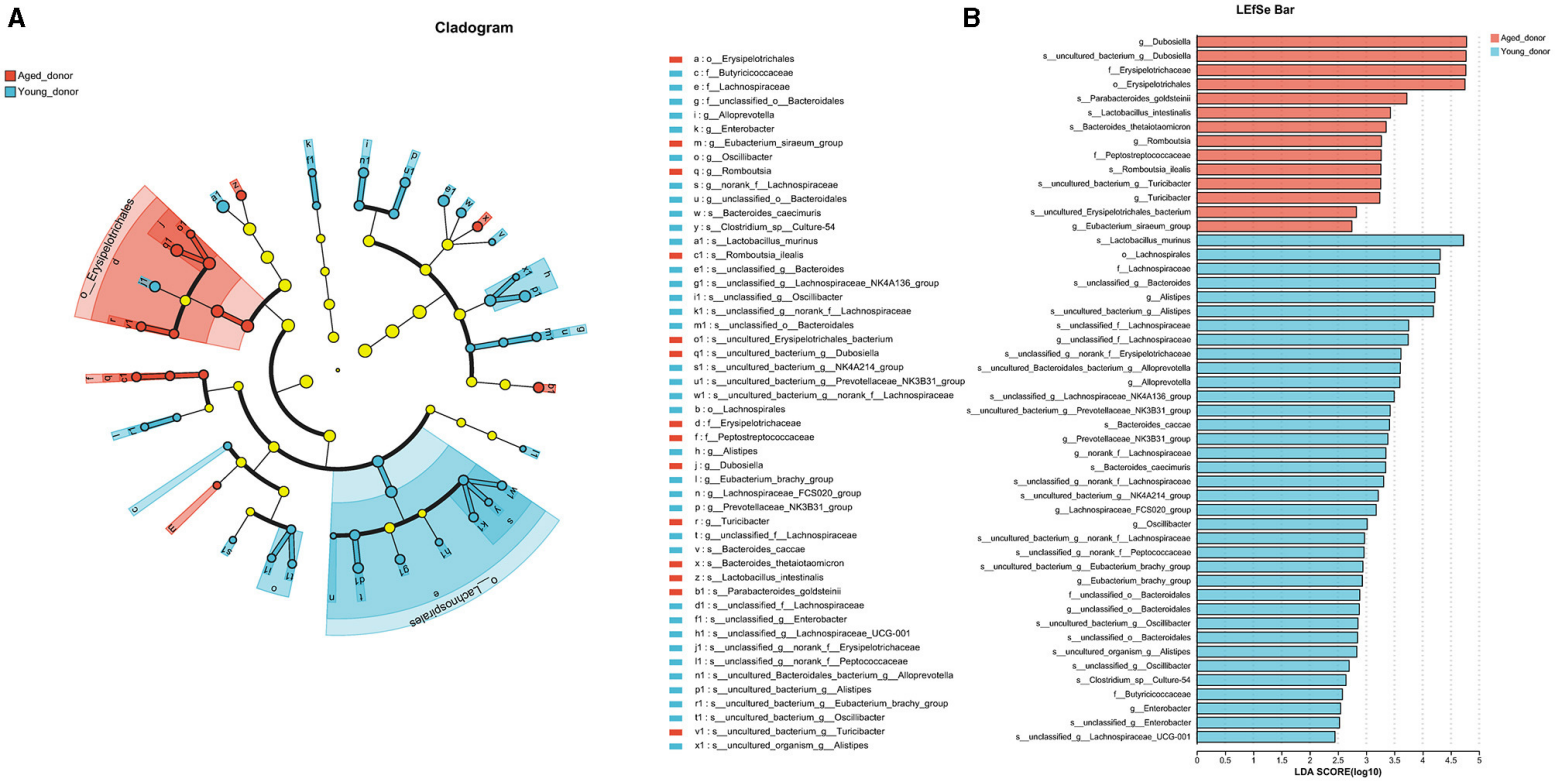
**(A)** The overall bacterial profiles of young and aged donor mice were represented by a LEfSe cladogram (*n* = 4 per group). This diagram highlights the taxonomic differences between the two groups, with each branch representing a specific bacterial lineage. **(B)** The LEfSe bar chart shows the relative abundance of bacteria from the phylum to genus level between aged and young donors. Only taxa with a significant LDA threshold value > 1 are listed (*n* = 4 per group). This chart illustrates which bacterial groups are more abundant in each age group, highlighting the key differences in gut microbiota composition between young and aged donor mice.

**Figure 6:**
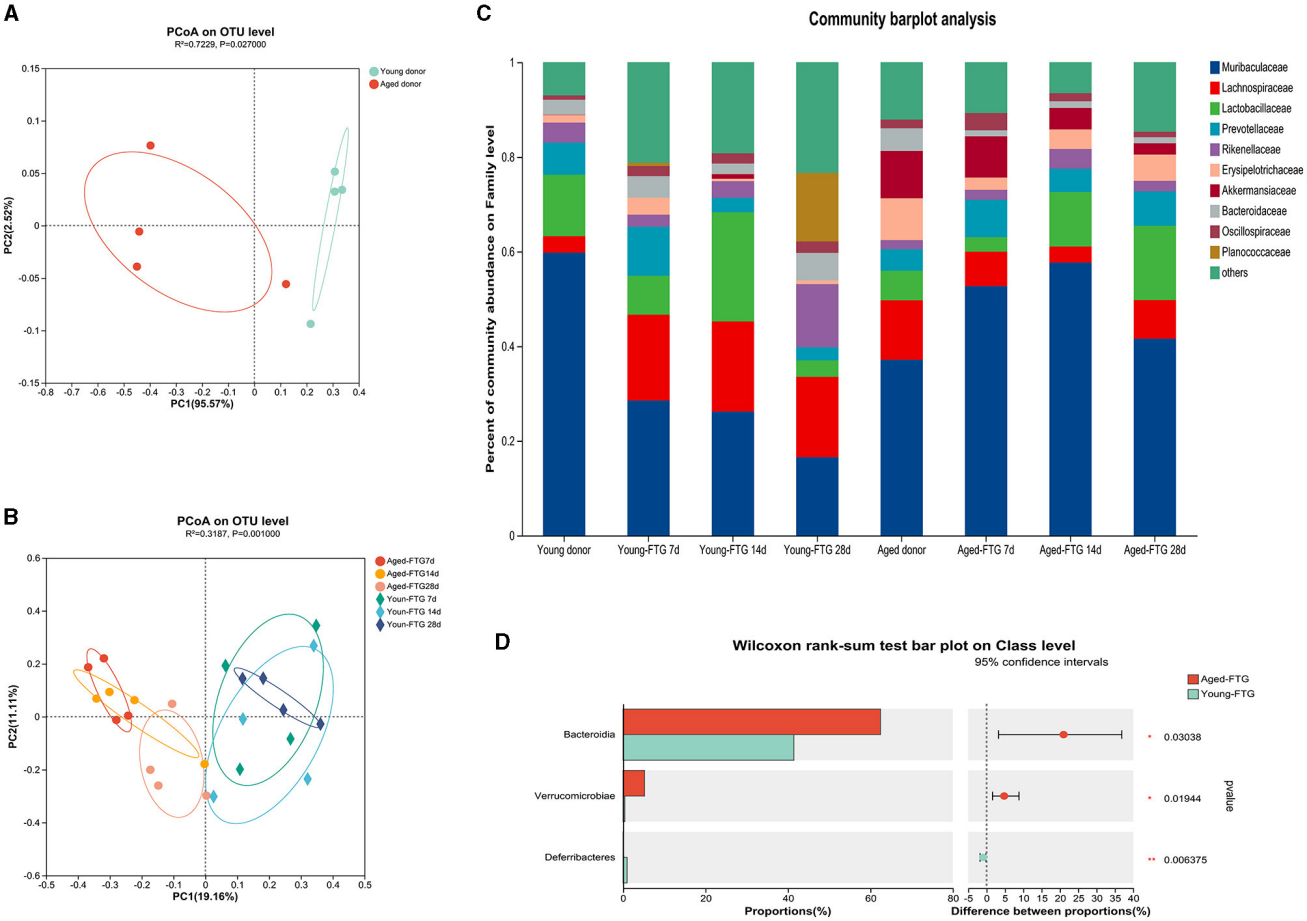
**(A)** Principal Coordinate Analysis (PCoA) of fecal microbiota from donor mice (young: 2–3 months, aged: 16–18 months) at the OTU level (R^2^ = 0.73, *P* = 0.027), *n* = 4 per group. This analysis illustrates the distinct separation between the microbiota of young and aged donors. **(B)** PCoA of fecal microbiota from FMT recipient mice at post-stroke days 7, 14, and 28 at the OTU level (R^2^ = 0.32, *P* = 0.001), *n* = 4 per group. This plot shows how the gut microbiota of the recipient mice clustered based on the age of the donor microbiome they received, with clear differences observed over time. **(C)** Community barplot analysis displaying the relative abundance of bacterial families in transplanted recipients on days 7, 14, and 28 post-dMCAO, compared to the original donors, *n* = 4 per group. This barplot provides a visual representation of how the gut microbiota composition in recipient mice reflects that of the donor mice over time. **(D)** The abundance of *Bacteroides* increased in the aged-FTG group compared with the young-FTG group at the class level (*P* = 0.03). This highlights a significant difference in the microbiome composition between recipient mice that received microbiota from young vs. aged donors.

We then profiled the gut bacterial composition using 16S rRNA sequencing. First, the gut microbiota composition between young and aged donor mice had significant differences, as highlighted by the LEfSe analysis cladogram (Figure 5A) and β-diversity analysis (*P* = 0.027, Figure 6A). Specifically, we found that the gut microbiota of young donors was enriched with *Lactobacillus* and *Lachnospiraceae*, both members of the *Firmicutes* phylum (Figure 5B). Conversely, the microbiota of aged donors was enriched with *Erysipelotrichaceae*, a family known to contain common pathogenic bacteria (Figure 5B).

Importantly, PCoA plots revealed that the fecal microbiome of recipient mice after FMT reflected that of their respective donors, with temporal changes observed on post-stroke days 7, 14, and 28 (*P* = 0.001, Figures 6B, C), which also proved that the FMT was effective. Aged stroke mice colonized with a young microbiome exhibited lower abundances of *Bacteroides*, which are known to include potential pathobionts (Gorvitovskaia et al., 2016), compared to those gavaged with aged FTG (Figure 6D).

The composition of the gut microbiota of young donor mice differed from that of older adults.

Aged recipient mice were colonized by young or aged microbiomes.

#### Young FMT enhances gut integrity in aged mice and increases acetate concentration in feces, serum, and cerebral cortex post-stroke

Given that the intestinal epithelial cell layer serves as a critical interface between gut microbiota and the brain, we sought to assess the regulatory effects of transplanted young microbiota on the intestinal barrier of aged stroke mice's colon. Intestinal tight junction proteins, such as Occludin and Claudins, play a vital role in maintaining intestinal barrier function and integrity (Furuse et al., 1998; Saitou et al., 2000). For a comprehensive evaluation, we compared the morphology and protein expression between the two groups.

**Figure 7:**
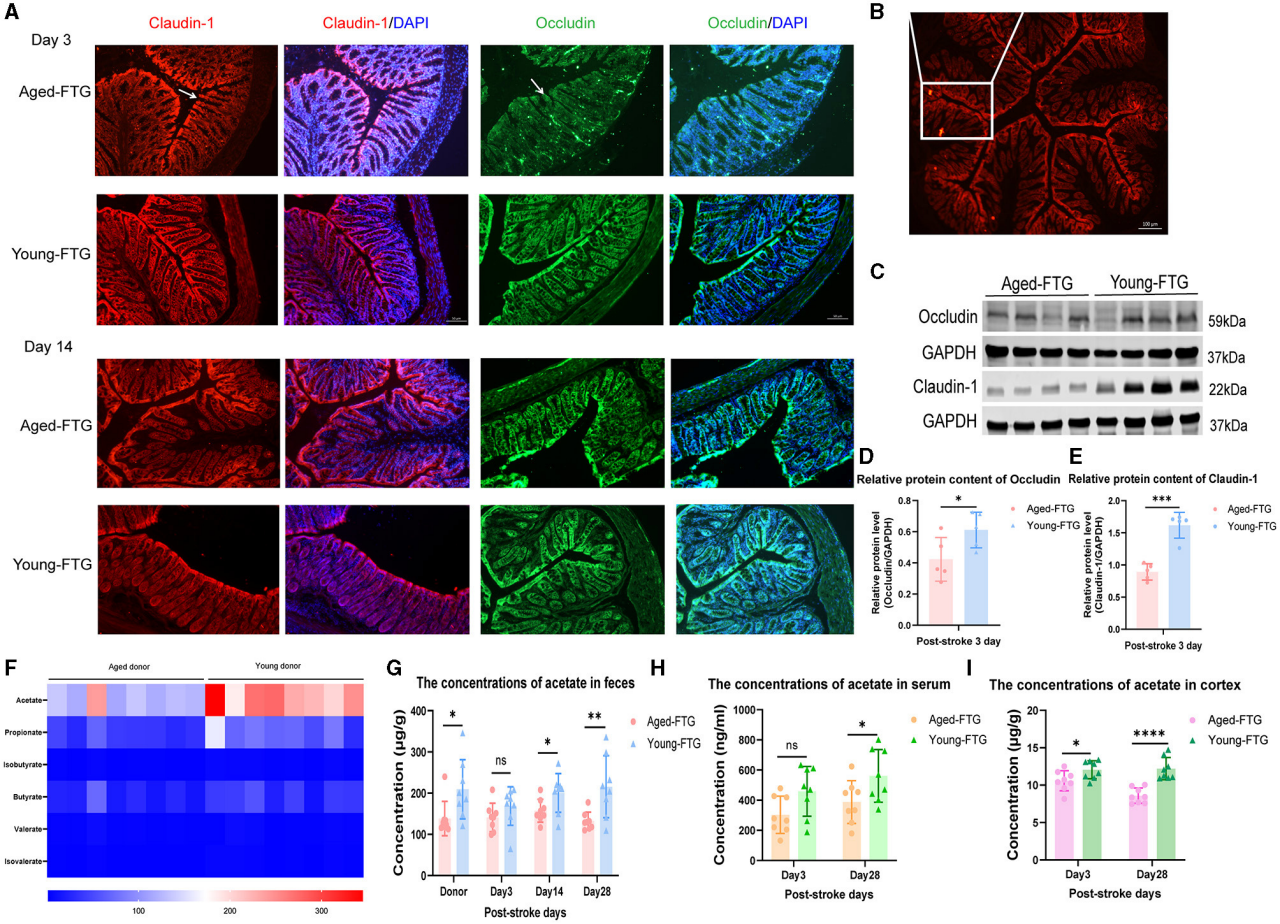
**(A, C)** Immunofluorescence staining of Claudin-1 and Occludin in the colon of aged stroke mice on post-MCAO days 3 and 14 (*n* = 4 per group). The arrows indicate the expression of Claudin-1 and Occludin. Scale bars for Claudin-1 and Occludin: 50 μm. **(B)** Schematic representation showing the locations where immunofluorescence images were taken. **(D, E)** Protein levels of Claudin-1 and Occludin in the colon of aged-FTG and young-FTG groups on day 3 post-stroke, respectively (*n* = 5 per group). **(F)** Heatmap showing the concentrations of SCFAs in young and aged donors (*n* = 8 per group). **(G–I)** Concentrations of fecal acetate, serum acetate, and brain cortex acetate in recipient mice. For two-group comparisons, Student's t-test was used after confirming data normality with the Shapiro-Wilk normality test **(C-E, G-I)**. Significance levels are indicated as follows: *p < 0.05, **p < 0.01, ***p < 0.001, ****p < 0.0001, comparing aged-FTG vs. young-FTG groups.

We found that the structure of intestinal barrier integrity was badly damaged in aged-FTG mice on post-stroke day 3 (Figure 7A), with Claudin-1 and Occludin barely visible in immunofluorescence (IF) images. Schematic representation showed the locations where immunofluorescence images were taken (Figure 7B). Although there was some recovery in the aged-FTG group by day 12, the young FTG group demonstrated a more rapid restoration of intestinal barrier integrity (Figure 7A). Similar changes were observed in the protein levels of Claudin-1 and Occludin in the colon, with these proteins being reduced in aged FTG mice compared to the young FTG on day 3 (Figures 7C–E). These findings suggest that transplantation with young microbiota is protective, improving defects in intestinal barrier integrity and reversing the altered expressions of tight junction protein.

Previous research showed that pre-treatment with a young microbiome, which contains higher levels of SCFAs, leads to better brain outcomes in recipient mice compared to an aged microbiome with lower SCFA levels (Lee et al., 2020a,b).

To this end, we first analyzed the SCFA concentrations in fecal samples used for FMT. Specifically, we measured SCFA concentrations, including acetate, propionate, and butyrate, in young donors (2–3 months) and aged mice (16–18 months). Mass spectrometry revealed an inverse relationship between aging and SCFA concentrations, with acetate levels significantly decreasing in aged mice (*P* = 0.0401) (Figure 7F), consistent with clinical results.

Subsequently, we measured fecal acetate concentrations in recipient mice on days 3, 14, and 28 post-stroke to assess the combined impacts of FTM and dMCAO (Figure 7G). Our study found statistically significant differences between the groups on post-stroke days 14 and 18 (*P* = 0.0422, *P* = 0.0092, Figure 7G). On day 3 post-stroke, the average concentration of fecal acetate decreased in the aged-FTG group, though the difference was not statistically significant (*P* = 0.2019, Figure 7G). We hypothesized that the cerebral ischemic model (dMCAO) had a greater effect on the mice. Finally, we also measured acetate levels in both serum and the cortex to determine whether acetate levels in the brain increased via the circulatory system (Figures 7H, I). The results revealed that acetate concentrations in the brain cortex increased in the young-FTG group from day 3 to day 28 post-stroke (Figure 7I). However, there was no significant difference in serum acetate levels on day 3, likely due to variability within the group (Figure 7I).

Young microbiota enhanced gut integrity in the epithelium of aged stroke mice and increased acetate concentration in feces, serum, and brain cortex.

#### Colonization of young microbiome promotes behavioral recovery (especially major muscle groups) and reduces infarct volume (acute phase) in aged stroke mice

**Figure 8:**
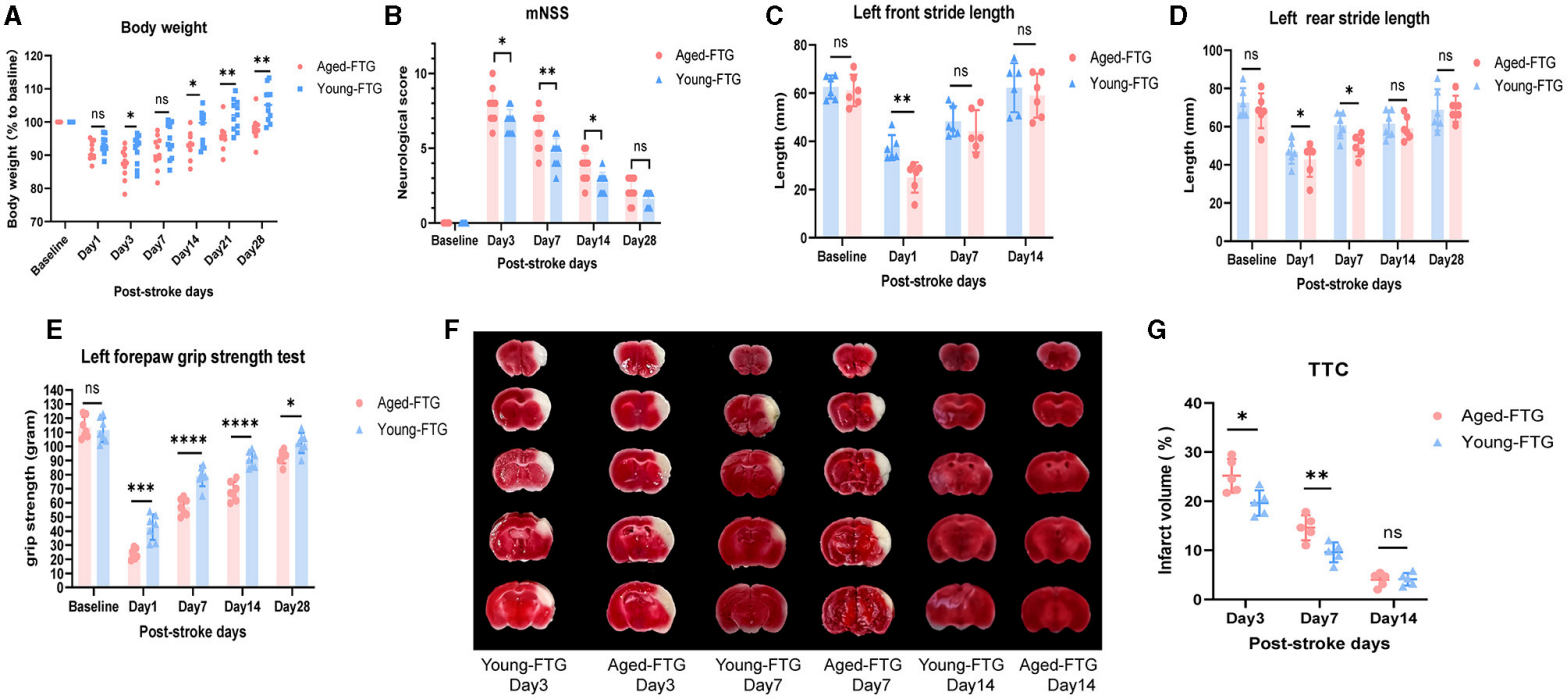
**(A)** Changes in body weight of young-FTG and aged-FTG mice on days 1, 3, 7, 14, 21, and 28 after cerebral ischemic, *n* = 11 per group. **(B)** Modified neurological severity score (mNSS) of the two groups, *n* = 10 per group. **(C–E)** Left front stride length **(C)**, left rear stride length **(D)**, and left forepaw strength **(E)** in young (*n* = 6) and aged FTG (*n* = 6) on day 14 following MCAO. **(F)** TTC-stained continuous brain sections of young-FTG and aged-FTG mice on days 3, 7, and 14 post-stroke. **(G)** The comparison of infarct volume between the two groups, *n* = 5 per group. Throughout, error bars represent mean ± SEM. For two-group comparisons, Student's t-test was used after confirming data normality using the Shapiro–Wilk normality test **(A–E, G)**. Significance levels are indicated as follows: *p < 0.05, **p < 0.01, ***p < 0.001, ****p < 0.0001 in the aged-FTG vs. young-FTG group.

Stroke patients and mice usually experience physiological changes such as weight loss (Crapser et al., 2016; Medeiros et al., 2020). To better assess the impact of a young microbiome, we evaluated changes in body weight in aged recipient mice on days 1, 3, 7, 14, 21, and 28 post-stroke (Figure 8A).

Mice in our study lost approximately 9% of their body weight by day 1 post-stroke, with no significant differences between groups (*P* = 0.9982). However, the aged-FTG group showed a lower minimum body weight compared to the young-FTG group on day 3 post-stroke. Over time, the young-FTG group recovered weight more quickly, and by day 21 after dMCAO, aged mice with a young microbiome had regained their pre-stroke body weight, whereas the aged-FTG mice had not returned to their original body weight by day 21 (*P* = 0.0165).

To evaluate whether young microbiota improved neurological function post-stroke, we performed a series of behavioral tests, including a modified neurological severity score (mNSS) to assess general neurological status, a grip strength test, and a gait test to evaluate muscle strength and coordination on the affected side. In the acute phase of stroke, mNSS scores significantly differed between the two groups (day 3 *P* = 0.0345, day 7 *P* = 0.0068, Figure 8B). By day 28 post-stroke, we did not observe a statistical significance in mNSS between the groups (*P* = 0.0594, Figure 8B).

Since the surgery was performed on the right cerebral arteries of the mice, it was particularly meaningful to observe gait and the strength of the left side. In the gait test, the front and rear limbs showed different recovery speeds in coordination and major muscle groups. Young-FTG mice performed better in both front and rear stride length (day 1: front, rear Figures 8C, D). By day 14 post-stroke, the front stride length had returned to baseline, while the rear stride length remained below baseline until day 28 post-stroke. Additionally, the grip strength test reflected subtle changes in functional analysis, particularly in the strength of smaller muscle groups. Aged stroke mice with young-FTG demonstrated increased left forepaw strength at all time points, although the difference between the two groups narrowed over time (Figure 8E).

The infarct volumes for each group on days 3, 7, and 14 post-stroke are shown in Figure 8F.

Consistent with the observed behavioral recovery, mice treated with young microbiota exhibited reduced infarct volumes compared to the aged-FTG group on post-stroke days 3 and 7 (day 3: *P* = 0.0109, day 7: *P* = 0.0256, Figure 8G). However, no statistical difference in infarct volume was observed between the two groups after 14 days (Figure 8F), which might be related to infarct atrophy.

Young microbiome improved post-stroke neurological function and infarct volume.

#### Young microbiota promotes long-term angiogenesis at three levels (cerebral blood flow, pial collateral vessels, and cortex microvessels) post-stroke in mice

We conducted experiments at three levels to gain a comprehensive understanding of the angiogenesis impacts of young microbiota.

**Figure 9:**
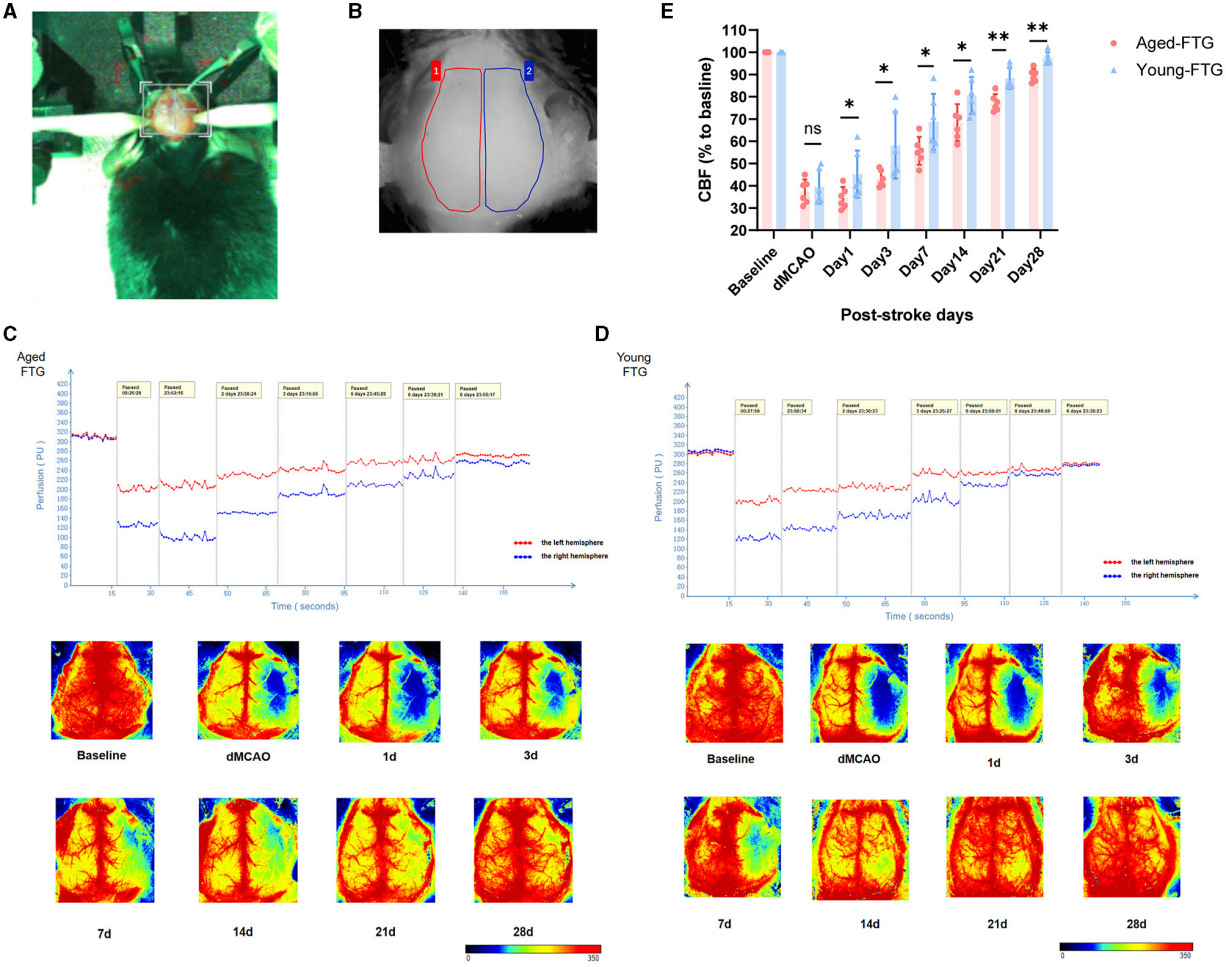
**(A)** The stereotaxic photo of the mouse cranial window. **(B)** The measured area of the right hemisphere (red color) and the left hemisphere (blue color, infarct side). **(C, D)** Quantification of CBF on both sides. CBF was measured using laser speckle imaging in aged-FTG and young-FTG groups on days 0, 1, 3, 7, 14, 21, and 28 after an ischemic stroke. *n* = 6 per group. **(E)** The statistical graph of CBF (*n* = 6). For two-group comparisons, the Student's *t-*test was used after confirming data normality with the Shapiro–Wilk normality test. Significance levels are indicated as follows: *P < 0.05, **P < 0.01 in the aged-FTG vs. young-FTG group.

First, we measured cerebral blood flow using laser speckle contrast imaging at various time points: baseline (before stroke), immediately post-surgery (dMCAO), and on days 1, 3, 7, 14, 21, and 28 post-stroke (Figures 9A–D). There was no statistical significance between the young-FTG and aged-FTG groups immediately after surgery (*P* = 0.5898, Figure 9E). However, by day 1 post-stroke, cerebral blood flow in the left hemisphere had decreased in most aged-FTG mice but began to recover in the young-FTG mice. From day 3 post-stroke, young microbiota significantly improved cerebral blood flow, with the young-FTG group showing better recovery than the aged-FTG group (day 3: *P* = 0.0377, day 7: *P* = 0.0456, day 14: *P* = 0.0292, day 21: *P* = 0.0015, day 28: *P* = 0.0008, Figure 9E).

**Figure 10:**
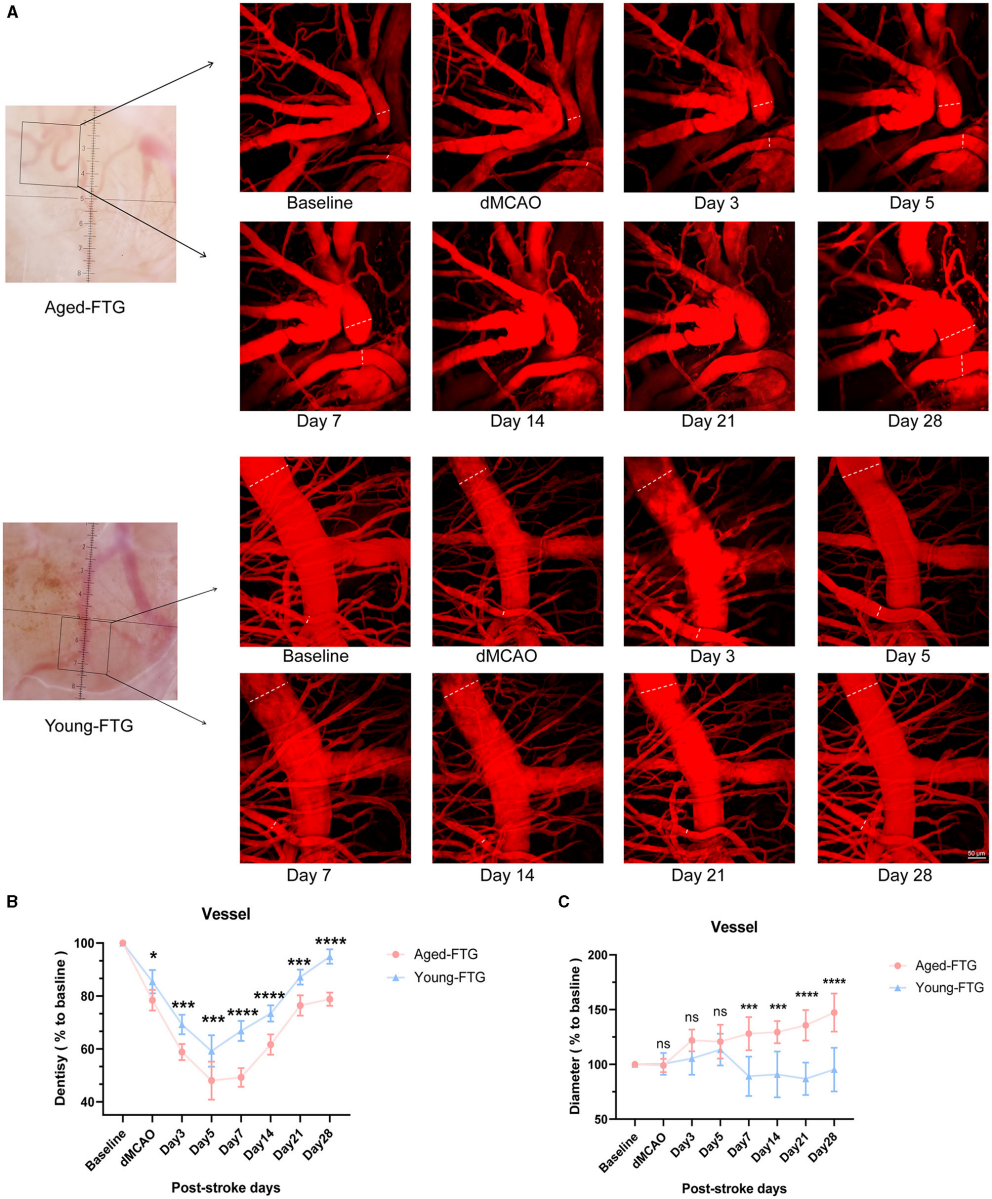
**(A)** White light images (left side) show surface images of the cortical brain through the cranial window at baseline. Black boxes indicate the areas magnified for 2-photon imaging. The enface maximum-intensity projections of 2-photon images within a 100 μm depth are shown for both groups at baseline and post-stroke (on days 3, 5, 7, 14, 21, and 28). The white line indicates collateral vessels used for quantitative analysis. **(B)** Quantitative analysis of pial collateral vessel density (*n* = 5). **(C)** Quantitative analysis of pial collateral vessel diameter (*n* = 5). Values are expressed as mean ± SEM (repeated-measures ANOVA). Significance levels are indicated as follows: *P < 0.05, ***P < 0.001, ****P < 0.0001, aged-FTG vs. young-FTG group.

As collateral remodeling status is a known key determinant of functional neurologic outcome post-stroke, we examined pial collateral remodeling between the two groups. Imaging was conducted to monitor the temporal dynamics of pial collateral remodeling post-stroke using *in vivo* 2-photon microscopy (Figure 10A). Pial collateral density and diameter were assessed as key indicators of collateral remodeling at each time point.

The vascular density of both groups began to decrease after surgery (Figure 10B). However, the vascular density in the aged-FTG group was consistently lower than in the young-FTG group from surgery through the end (dMCAO: *P* = 0.0392, day 3: *P* = 0.0004, day 5: *P* = 0.0001, day 7, 14, 28: *P* < 0.0001, Figure 10B).

On days 7, 14, 21, and 28 post-stroke, the pial collateral diameter was significantly larger in the ipsilateral hemisphere of the aged-FTG group (121.8% ± 15.12%, 129.4% ± 10.16%, 135.7% ± 13.84%, and 147.3% ± 17.41%) compared to the young-FTG group (89.14% ± 17.92%, 90.86% ± 20.90%, 86.85% ± 14.83%, and 95.27% ± 19.96%, Figure 10C). We suspected that the faster blood flow recovery in the young-FTG group led to the retraction of blood vessels.

**Figure 11:**
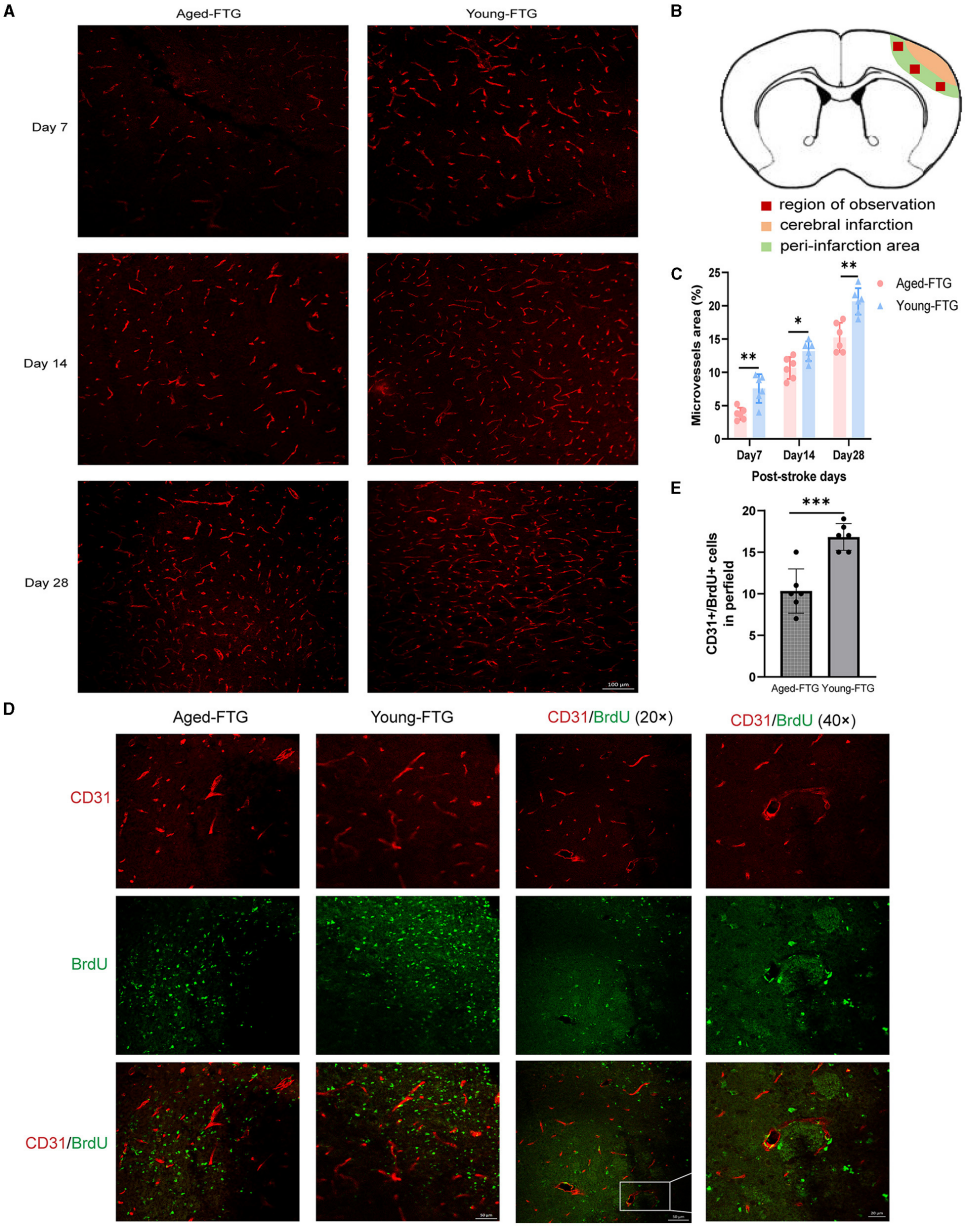
**(A)** Representative immunofluorescence images of CD31-labeled microvessels in the young-FTG and aged-FTG groups on days 7, 14, and 28 after cerebral ischemia. Scale bar: 100 μm. **(B)** In the schematic brain diagram, the orange area represents the infarct area. Red boxes indicate the peri-infarct cortex of the ipsilateral hemisphere, where immunofluorescence images were taken. **(C)** Quantification of microvascular density (*n* = 6 per group). **(D)** Immunofluorescent staining of BrdU (green)/CD31 (red) cells and microvessels in the peri-infarct cortex on day 7 post-stroke (the first two columns). Scale bar: 50 μm. The last two columns show magnified area for BrdU+/CD31+ microvessel endothelial cells. Scale bar: 20 μm. **(E)** Quantification of BrdU+/CD31+ cells in the peri-infarct cortex on day 7 post-stroke (*n* = 6 per group). *P < 0.05, ** indicates *P* < 0.01, *** indicates *P* < 0.001 in the aged-FTG vs. young-FTG group.

Finally, the cortical penumbra microvessels were investigated using immunofluorescence co-localization analysis to determine the effect of young microbiota (Figure 11B). We examined the microvessel density in the peri-infarction area using CD31 staining.

On day 7 post-stroke, microvessels began to increase and continued to increase through days 14 to 28 (Figure 11A). Our data indicated that the microvessel density in the young-FTG group was higher than that in the aged-FTG group (day 7: *P* = 0.026; day 14: *P* = 0.0192, day 28: *P* = 0.0012, *n* = 6 each group, Figure 11C).

We also examined the co-localization of BrdU+/CD31+ in the microvessels to assess the distribution of angiogenesis on day 7 post-stroke (Figure 11D). The number of endothelial cells in the young-FTG group was higher than in the aged-FTG group (*P* = 0.0004, Figure 11E).

**Figure 12:**
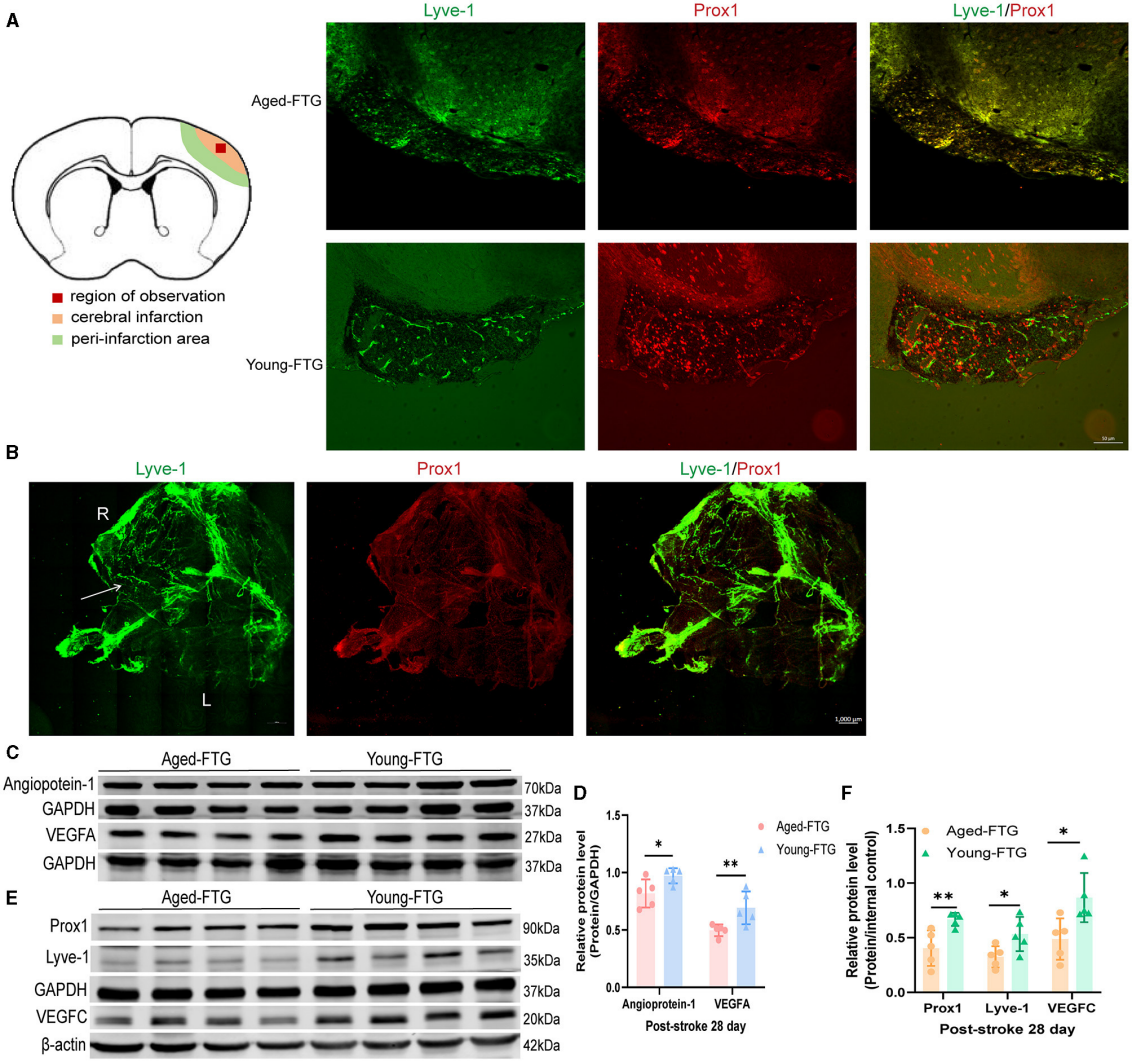
**(A)** In the schematic brain diagram, the orange area represents the infarct area. Red boxes indicate the regions where immunofluorescence images were taken. The left side shows the ingrowth of lymphatic vessels on day 28, past stroke. Scale bar: 50 μm. **(B)** The distribution of meningeal lymphatic vessels on day 3 post-stroke in young FTG mice. The white arrow indicates the growth of lymphatic vessels. R: right side, L: left side. Scale bar: 1,000 μm. **(C)** Representative Western blot images of VEGF/GAPDH and Ang1/GAPDH in the cortex on day 28 post-stroke. **(D)** Quantification of VEGF/GAPDH and Ang1/GAPDH relative expression on day 28 post-stroke (*n* = 5 per group). **(E)** Representative Western blot images of PROX1/GAPDH, Lyve-1/GAPDH, and VEGFC/β-actin in the cortex on day 28 post-stroke (*n* = 5 per group). **(F)** Quantification of PROX1/GAPDH, Lyve-1/GAPDH, and VEGFC/β-actin relative expression on day 28 post-stroke.*P < 0.05, ** indicates a *p-*value of <0.01 in the aged-FTG vs. young-FTG group.

We performed western blotting on the cortical penumbra to assess angiogenesis. Compared to the aged-FTG group, the young microbiota FMT group showed increased protein expression of VEGFA on day 28 (*P* = 0.0205, *n* = 5 per group, Figures 12C, D). The protein expression level of Ang1 was also increased in the young-FTG group (*P* = 0.0384, *n* = 5 each group, Figures 12C, D).

Young FMT improved cerebral blood flow after ischemic stroke.

Young FMT increased pial collateral remodeling after ischemic stroke *in vivo* 2-photon imaging.

Young FMT increased cortical penumbra microvessels in immunofluorescence post-stroke.

#### Young microbiota promotes lymphatic ingrowth in cerebral infarction in aged stroke mice

To explore the mechanism by which the young microbiome affects cerebral angiogenesis, we examined the distribution of meningeal lymphatic vessels and the presence of lymphatic ingrowth in the cerebral cortex. We successfully observed the temporal dynamics of lymphatic vessels. In the young-FTG group, an increase in meningeal lymphatic vessels was noted in the right (ipsilateral) hemisphere on day 3 post-stroke (Figure 12B). However, lymphatic vessel ingrowth into the cerebral cortex was not observed at this stage. By day 28 post-stroke, lymphatic vessels had wrapped around the infarct area, resembling a cocoon in the young-FTG group, a formation not observed in the aged-FTG group (Figure 12A). Finally, we quantitatively measured lymphatic protein levels in the right cerebral cortex to assess lymphatic ingrowth. Lymphangiogenesis is dependent on VEGFC (Villefranc et al., 2013) and is characterized by the drainage of interstitial fluid and the expression of molecular markers, including Prox1, Vegfr3/Flt4, and Lyve1 (Flores et al., 2010; Okuda et al., 2012). Compared with the aged-FTG group, the protein expression of VEGFC, Lyve-1, and PROX1 was significantly increased in the young microbiota FMT group on day 28 (*P* = 0.0205, *P* = 0.0355, *P* = 0.0092, *n* = 5 per group, Figures 12E, F).

Young FMT promoted lymphatic ingrowth in aged stroke mice.

## Discussion and conclusion

In this study, we found that the gut microbiota from “young donors” promoted angiogenesis in both older stroke patients and aged stroke mice. Among older stroke patients, the diversity of the gut microbiota was significantly reduced compared to young stroke patients. Furthermore, acetate, a bacterially derived SCFA, was decreased and positively correlated with angiogenesis markers, such as VEGF and VEGF-C.

In aged stroke mice, transplantation of young microbiota improved stroke outcomes through enhanced angiogenesis and lymphatic ingrowth. This protective effect was attributed to gut microbiota-derived acetate, which promoted lymphangiogenesis by replenishing acetyl coenzyme A (Wong et al., 2017).

As the global population ages, with those over 60 expected to comprise 22% of the population by 2050 (Zhou et al., 2022), the prevalence and morbidity of aging-related diseases, including stroke, are likely to rise. Stroke, a common condition in older adults, has become a major focus in neurology.

Previous research by Yamashiro et al. (2017) demonstrated a decrease in acetate levels in the fecal samples of stroke patients, highlighting the need to understand the unique impact of environmental factors on functional recovery in the elderly stroke population.

Moreover, our study revealed that younger stroke patients had higher levels of beneficial gut bacteria such as *Bifidobacterium, Faecalibacterium*, and *Lactobacillus* compared to their older counterparts. This led us to explore how the gut microbiota influences the brain, focusing on bacterial-derived SCFAs—key molecules in the gut-brain axis. Therefore, we assessed the concentration of SCFAs in fecal samples to investigate their role further.

Interestingly, the concentration of acetate decreased significantly in older stroke patients compared to younger ones. Current main treatments for ischemic stroke include intravenous thrombolysis within the therapeutic time window, antiplatelet therapy, and endovascular thrombectomy (Herpich and Rincon, 2020).

However, few treatments target angiogenesis, which is a crucial restorative mechanism that responds to cerebral ischemia by promoting tissue repair, vascular remodeling, and plasticity (Sun et al., 2021). The vascular endothelial growth factor (VEGF) family has five isoforms, including proangiogenic factors (VEGF-A, VEGF-B) (Liu et al., 2023), pro-lymphangiogenic factors (VEGF-C and VEGF-D) (Detoraki et al., 2009), and placental growth factor. Among these, VEGF-A is the most potent isoform for angiogenesis, binding to two structurally related receptor-type tyrosine kinases in vascular endothelial cells (Ferrara et al., 2003; Shibuya, 2013; Abhinand et al., 2016).

Cobbs showed that VEGF-A is significantly upregulated in the ischemic penumbra region after focal cerebral ischemia (Cobbs et al., 1998). In this study, we focused on VEGF-A, referring to it as VEGF.

Lymphangiogenesis, the process by which lymphatic endothelial cells (LECs) are formed, occurs through the budding and differentiation of original endothelial cells into LECs (Wang et al., 2023). The PROX1/VEGF-C/VEGFR3 pathway is fundamental to this process (Mauri et al., 2018; Oliver et al., 2020).

Further analysis revealed that acetate positively correlated with VEGF and VEGF-C, both of which are key indicators of angiogenesis. In the clinical trial, achieving ideal control over a single variable is challenging due to the variability in patients' diets and lifestyle habits, genotype, and stroke severity. The correlation analysis showed that most correlation coefficients were around 0.3, indicating a weak positive correlation, which might also be influenced by clinical trial biases. Nonetheless, these findings encouraged us to further investigate the relationship between gut microbiota and angiogenesis.

In animal experiments, most studies on stroke in aged individuals primarily focus on neuroinflammation. For instance, Gresita et al. (2022) showed that early exposure to a stimulating social, sensory, and motor environment effectively improved behavioral recovery (measured by rotating pole and working memory tasks) in aged stroke mice and was associated with reduced neuroinflammation.

Similarly, Lee et al. (2020a,b) found that young FMT reduced pro-inflammatory IL-17 levels in brain γδT cells in aged stroke mice. However, few studies have explored the relationship between angiogenesis and stroke in older adults.

In our study, we were the first to demonstrate that gut microbiota-derived acetate improved stroke outcomes through angiogenesis and lymphatic ingrowth in aged stroke mice. We found that transplanting young microbiota into aged mice had several beneficial effects on behavioral recovery (as evidenced by improvements in mNSS, gait tests, and grip strength).

While previous research by Lee et al. (2020a,b) found no differences in infarct size between FTG groups post-stroke day 14, our study shortened the time intervals and provided a more comprehensive conclusion. We observed that mice treated with young microbiota exhibited reduced infarct volumes on post-stroke days 3 and 7 compared to the aged-FTG group, although no statistical difference was observed at 14 days post-stroke. This protective effect was linked to the enhancement of gut barrier integrity and angiogenesis in the brain cortex.

The gut epithelium, composed of a single layer of epithelial cells, is crucial for maintaining homeostasis and mucosal immunity, playing an integral role in the dialogue with luminal bacteria (Artis, 2008). The intestinal barrier consists of three main components: the mucus layer, the intestinal epithelium, and the immune defense mechanisms that extend from the intestinal lumen to the muscle layer.

The physical–mechanical barrier of the intestine includes various cellular structures, including adhesion junctions and tight junctions (TJs). TJs consist of several proteins, including claudins, occludin, zonula occludens-1, and junctional adhesion molecule-1, all of which are essential for maintaining intestinal barrier function and integrity (Furuse et al., 1998; Saitou et al., 2000).

Following dMCAO, the expression of Claudin-1 and Occludin at the edges of intestinal epithelial cells appeared intermittently or discontinuously in immunofluorescence staining. We found that the intestinal barrier integrity of the aged-FTG group mice was severely damaged on day 3 post-stroke (Figure 7A). Although the gut condition of the aged-FTG group improved after 11 days, the intestinal barrier integrity of the young-FTG group recovered more rapidly (Figure 7A). These findings indicate that young FMT enhanced intestinal integrity by upregulating intestinal TJ proteins (Occludin and Claudin-1) and increasing the number of mature goblet cells in the upper crypts. The enhancement of integrity is also associated with the increased expression of intestinal tight junction proteins, such as occludin and claudins.

We explored the possible mechanism by which young gut microbiota influence cerebral angiogenesis after ischemic stroke. First, we found that young microbiota indeed promoted cerebral angiogenesis following dMCAO in aged mice. We verified our hypothesis at three different levels: hemispheric cerebral blood flow, lateral cerebral vessels, and cerebral cortical microvessels.

The young-FTG group did not experience the transient decline in cerebral blood flow observed on day 1 after dMCAO and showed a faster recovery in hemispheric cerebral blood flow compared to the aged-FTG mice. In the lateral cerebral vessels of the meninx, both groups experienced a progressive decrease in vessel density following dMCAO. However, in the Young-FTG group, vessel density began to recover by day 7 post-dMCAO.

Meanwhile, vascular density in the aged-FTG group remained lower compared to day 5 post-dMCAO, indicating a slower recovery. In contrast, the Young-FTG group demonstrated a stronger recovery in both vascular density and diameter. Observations of microvessels in the ischemic penumbra of the cerebral cortex supported these findings, suggesting that gut microbiota promotes cerebral vascular regeneration after focal cerebral infarction in young mice.

What are the possible mechanisms behind this?

In our clinical trial, we found that fecal acetate levels were higher in young stroke patients compared to aged stroke patients. Coincidentally, the transplantation of young gut microbiota into aged mice also led to an increase in fecal acetate content in the animal experiment. We also detected increased acetate levels in the serum and cerebral cortex, which indicated that bacteria-produced acetate was able to cross the blood–brain barrier and reach brain tissue. Wong et al. found that *in vivo* supplementation of acetic acid can restore acetyl-coA levels, which is used by histone acetyltransferase p300 to acetylate histones on lymphangiogenesis genes (Wong et al., 2017). In other words, supplementing with acetic acid promotes the formation of lymphatic vessels.

Lymphatic vessels (LVs), lined by lymphatic endothelial cells (LECs), play essential roles in fat absorption, fluid homeostasis, and several pathological processes, including lymphedema and tumor metastasis (Alitalo et al., 2005). Lymphatic vessel endothelial hyaluronan receptor 1 (LYVE1), a receptor of LECs, is also involved in regulating lymphangiogenesis. For a long time, it was believed that the central nervous system lacked lymphatic vasculature. However, recent discoveries have identified meningeal lymphatics in the mouse endocranium as authentic lymphatic vessels that drain cerebrospinal fluid into the periphery (Aspelund et al., 2015; Louveau et al., 2015).

Moreover, the roles of LVs in reducing edema and promoting cerebral angiogenesis after cerebrovascular damage have recently gained attention (Chen et al., 2019; Yanev et al., 2020; Chen et al., 2021). While the brain parenchyma typically lacks mammalian lymphatics under physiological conditions (Chen et al., 2019), Chen et al. (2019) found that photothrombotic-induced lymphatic invasion into the brain parenchyma could guide vascular regeneration in cerebrovascular injuries, as observed in zebrafish.

Additionally, Yanev et al. (2020) discovered photothrombosis-induced meningeal lymphangiogenesis in young male mice, though not in cases of transient middle cerebral artery occlusion (tMCAO).

Until now, there have been no reports on whether LVs invade the brain parenchyma in stroke mice to facilitate angiogenesis. Our study is the first to reveal that young microbiota promote meningeal lymphangiogenesis, lymphatic ingrowth into the infarcted area, and angiogenesis in mice post-dMCAO.

How did gut microbiota induce lymphangiogenesis?

*In vivo*, acetate supplementation can rescue the process of replenishing acetyl coenzyme A, which is used by histone acetyltransferase p300 to acetylate histones at lymphangiogenic genes (Wong et al., 2017). We observed an increase in gut microbiota-derived acetate in the feces, serum, and the cerebral cortex of young FTG mice. Therefore, we hypothesized that young microbiota significantly improves stroke outcomes by inducing lymphangiogenesis, promoting tissue fluid absorption in the infarcted area, and facilitating angiogenesis in the infarcted hemisphere. This supports the idea that the invasion of LVs to guide angiogenesis in the young microbiota group, where SCFA-producing bacteria (probiotics) are more likely to produce acetate than in the aged-FTG group, plays a critical role in improving stroke outcomes. Notably, these effects appear to be more closely related to the “composition” of the microbiota than to the chronological age of the mice.

In conclusion, our study comprised two key components: a clinical trial and an animal experiment. The clinical trial revealed significant differences in the microbiota of older stroke patients compared to younger ones. Notably, probiotic-derived acetate was reduced in older stroke patients and showed a positive correlation with angiogenesis markers (VEGF and VEGF-C). To achieve more controlled results, we transplanted young gut microbiota into aged mice, which enhanced stroke recovery through angiogenesis driven by meningeal lymphangiogenesis and lymphatic ingrowth. This process was primarily mediated by bacterially produced metabolites, particularly SCFAs such as acetate.

Our findings also indicated that young microbiota significantly improved gut integrity in aged stroke mice. This study is the first to provide direct clinical and experimental evidence that (a) gut microbiota-derived acetate promotes angiogenesis post-stroke and (b) lymphatic ingrowth occurs in the cerebral cortex of the mice post-dMCAO. Our research strongly supports the concept that targeting “bottom-up signaling” from the gut to the brain can enhance post-stroke recovery in aged individuals, broadening our understanding of the gut-brain axis. These findings suggest that selectively targeting SCFA-producing bacteria (such as acetate-producers) could offer a promising protective and therapeutic strategy to mitigate functional impairments in aged stroke patients. Harnessing the therapeutic potential of microbiota composition could be a key approach to improving recovery post-stroke.

## Data Availability

The raw sequence data reported in the study are deposited in the Genome Sequence Archive (Genomics, Proteomics & Bioinformatics 2021) in National Genomics Data Center (Nucleic Acids Res 2022), China National Center for Bioinformation / Beijing Institute of Genomics, Chinese Academy of Sciences (GSA: CRA016453) that are publicly accessible at https://ngdc.cncb.ac.cn/gsa.

## Supplementary material

For the 16S rRNA sequencing, the generated sequences were clustered at 97% similarity to form out, which revealed the composition of the intestinal flora community. The Sobs and Shannon diversity rarefaction curves plateaued, showing that the sequence data were sufficient and accurately represented the community.

Supplementary Figure S1 **(A)** Alpha diversity estimators calculated indexes of Sobs on the OTU level. **(B, C)** The rarefaction curve calculated the indexes of Shannon and Sobs at the OTU level.

Supplementary Figure S2 **(A)** The community composition of the two groups was analyzed at the genus level. **(B)** The Circos graph showed the most abundant categories of the gut microbiota. This visual circular diagram showed the species-sample correspondence, displaying which microbes were present in each sample (or group) and their relative abundance. **(C)** Functional prediction analysis of bacterial microbiota was conducted using PICRUSt2 combined with the KEGG database to predict the function profile of the bacterial microbiota in tissues. The results, shown at KEGG Level 2, indicated that the function of gut microbiota between the groups was related to aging and the nervous system. Gut microbiota changes in recipient-aged mice (StM) compared with normal-aged mice (Ctrl): after the mice were gavaged with streptomycin treatment, the α-diversity and β-diversity significantly decreased in the StM group. This indicated a reduction in bacterial abundance and diversity in fecal samples after 2 consecutive days of streptomycin treatment.

Supplementary Figure S3 **(A)** Alpha diversity estimators calculated the Ace index at the OTU level. *n* = 8 per group. **(B)** Alpha diversity estimators calculated the Chao index at the OTU level. *n* = 8 per group. **(C)** The rarefaction curve calculated the Sobs index at the OTU level. *n* = 8 per group. **(D)** The rarefaction curve calculated the Shannon index at the OTU level. *n* = 8 per group. **(E)** Beta diversity estimators calculated the PCoA on the OTU level. *n* = 4 per group.
