# Supplementary figures and images for: Gut microbiota-derived acetate promotes long-term recovery through angiogenesis guided by lymphatic ingrowth in older adults with stroke
Source: Front Neurosci. 2024 Sep 20;18:1398913. doi: 10.3389/fnins.2024.1398913 (PMC11450648; doi:10.3389/fnins.2024.1398913)

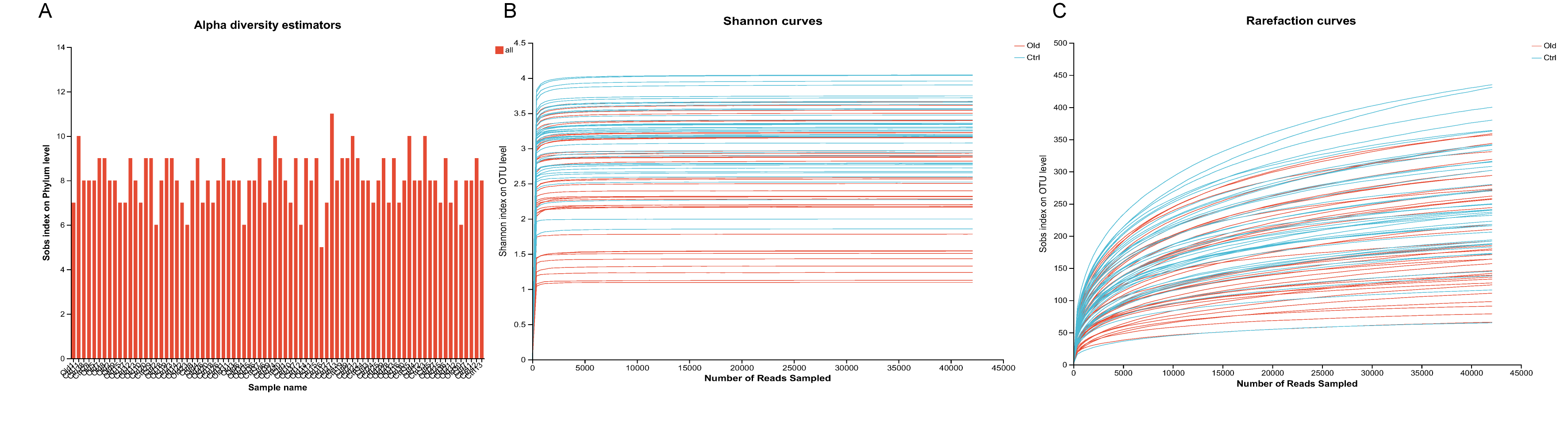

Supplement: Supplementary Figure S1 — (A) Alpha diversity estimators calculated indexes of Sobs on the OTU level. (B, C) The rarefaction curve calculated the indexes of Shannon and Sobs at the OTU level. [file Image_1.tif]

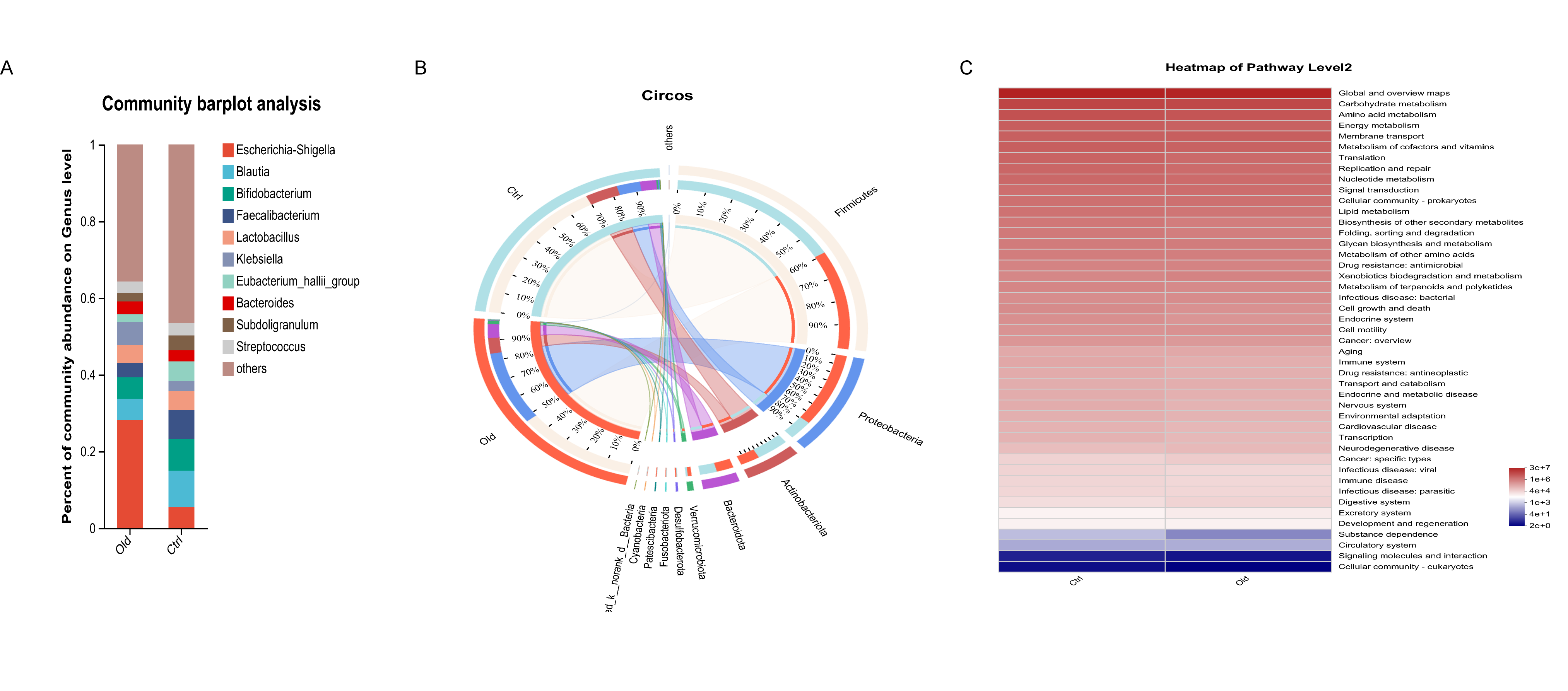

Supplement: Supplementary Figure S2 — (A) The community composition of the two groups was analyzed at the genus level. (B) The Circos graph showed the most abundant categories of the gut microbiota. This visual circular diagram showed the species-sample correspondence, displaying which microbes were present in each sample (or group) and their relative abundance. (C) Functional prediction analysis of bacterial microbiota was conducted using PICRUSt2 combined with the KEGG database to predict the function profile of the bacterial microbiota in tissues. The results, shown at KEGG Level 2, indicated that the function of gut microbiota between the groups was related to aging and the nervous system. Gut microbiota changes in recipient-aged mice (StM) compared with normal-aged mice (Ctrl): after the mice were gavaged with streptomycin treatment, the α-diversity and β-diversity significantly decreased in the StM group. This indicated a reduction in bacterial abundance and diversity in fecal samples after 2 consecutive days of streptomycin treatment. [file Image_2.tif]

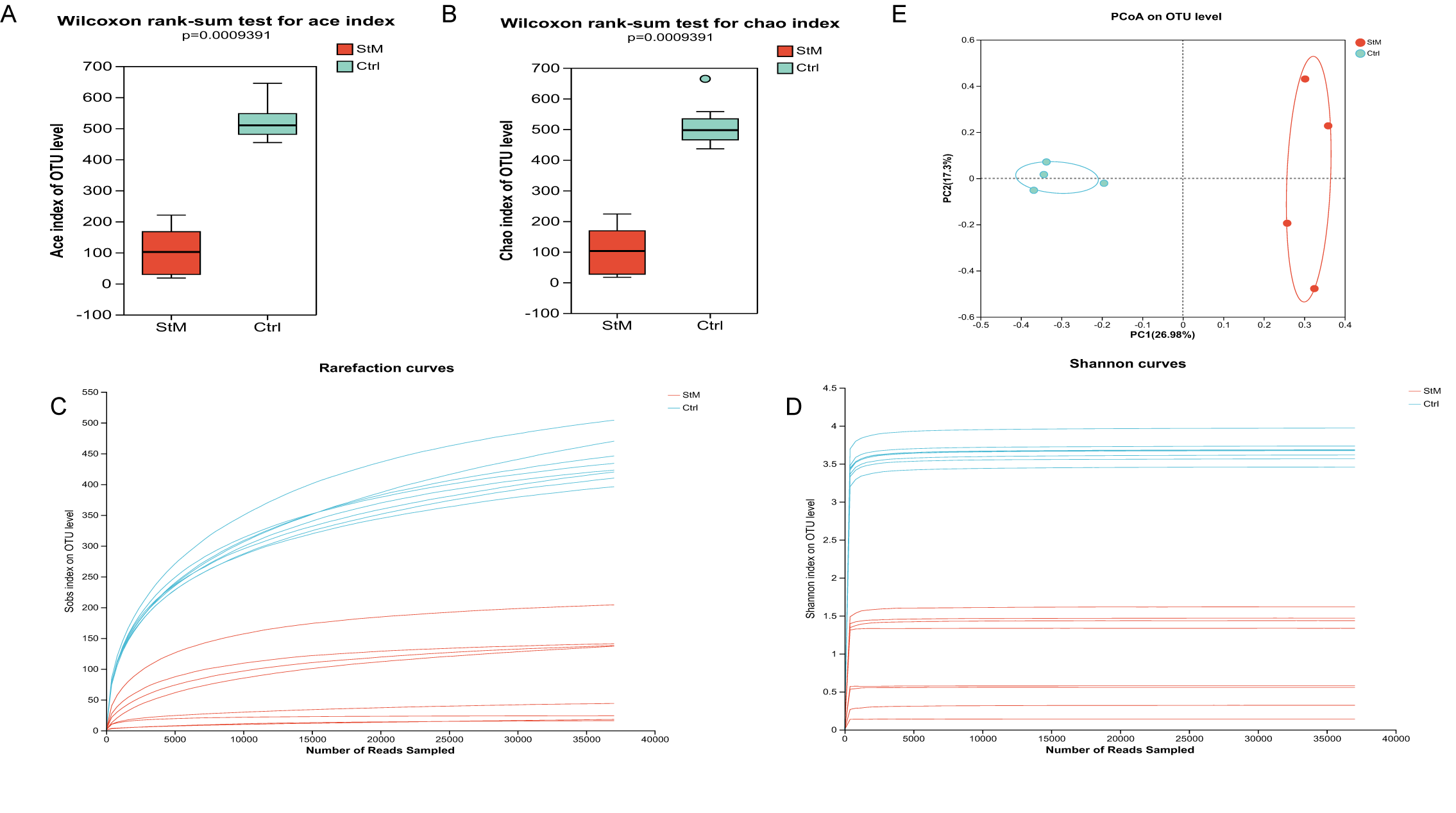

Supplement: Supplementary Figure S3 — (A) Alpha diversity estimators calculated the Ace index at the OTU level. n = 8 per group. (B) Alpha diversity estimators calculated the Chao index at the OTU level. n = 8 per group. (C) The rarefaction curve calculated the Sobs index at the OTU level. n = 8 per group. (D) The rarefaction curve calculated the Shannon index at the OTU level. n = 8 per group. (E) Beta diversity estimators calculated the PCoA on the OTU level. n = 4 per group. [file Image_3.tif]
